# The secreted ribonuclease T2 protein FoRnt2 contributes to *Fusarium oxysporum* virulence

**DOI:** 10.1111/mpp.13237

**Published:** 2022-06-13

**Authors:** Hengwei Qian, Lulu Wang, Baoshan Wang, Wenxing Liang

**Affiliations:** ^1^ College of Life Sciences Shandong Normal University Jinan China; ^2^ Key Lab of Integrated Crop Pest Management of Shandong Province College of Plant Health and Medicine, Qingdao Agricultural University Qingdao China

**Keywords:** *Fusarium oxysporum*, ribonucleases, secreted protein, virulence

## Abstract

Secreted RNase proteins have been reported from only a few pathogens, and relatively little is known about their biological functions. *Fusarium oxysporum* is a soilborne fungal pathogen that causes Fusarium wilt, one of the most important diseases on tomato. During the infection of *F. oxysporum*, some proteins are secreted that modulate host plant immunity and promote pathogen invasion. In this study, we identify an RNase, FoRnt2, from the *F. oxysporum* secretome that belongs to the ribonuclease T2 family. FoRnt2 possesses an N‐terminal signal peptide and can be secreted from *F. oxysporum*. FoRnt2 exhibited ribonuclease activity and was able to degrade the host plant total RNA in vitro dependent on the active site residues H80 and H142. Deletion of the *FoRnt2* gene reduced fungal virulence but had no obvious effect on mycelial growth and conidial production. The expression of *FoRnt2* in tomato significantly enhanced plant susceptibility to pathogens. These data indicate that FoRnt2 is an important contributor to the virulence of *F. oxysporum*, possibly through the degradation of plant RNA.

## Graphical abstract

FoRnt2, a member of the ribonuclease T2 family secreted from *Fusarium oxysporum*, is able to degrade host RNA and represents an important contributor to the virulence of the soilborne fungal pathogen.

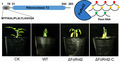

## 1 INTRODUCTION

Plant pathogens can directly enter plant epidermal cells or extend their hyphae into plant cells to promote the development of pathogens and the occurrence of disease (Jones & Dangl, 2006). The defence or immune response offered by multilayered physical barriers helps host plants defeat invading pathogens (Cao et al., 2018; Ingle et al., 2006; Mengiste, 2012; Postel & Kemmerling, 2009). However, fungal pathogens can secrete several proteins in host cells to suppress plant immune responses to effectively infect the hosts. The secreted proteins function mainly in several physiological and pathological processes such as RNA silencing and cell signal transduction, and interfere with plant cellular metabolism (Krause et al., 2013; Sharpee & Dean, 2016). Most of these proteins are secreted extracellularly through the endoplasmic reticulum (ER)/Golgi pathway, which requires a hydrophobic signal peptide at the N‐terminus of proteins for translocation (Prudovsky et al., 2008). Pathogenic oomycetes can secrete some effector proteins, which share RxLR, CRN or ChxC amino acid sequence motifs, in host plant cells (Yang et al., 2017), and bacteria deliver effector proteins to the host using the type III secretion system (Choi et al., 2017). Unlike the effectors from bacteria and oomycetes, a small group of effectors from wheat leaf rust and barley powdery mildew possess a conserved Y/F/WxC motif (Godfrey et al., 2010). However, most of the proteins secreted by pathogenic fungi in hosts have no conserved sequence motif (Yang et al., 2021).

Pathogens secrete some cell wall‐degrading enzymes to disrupt the cell wall for infection (Blackman et al., 2014). For example, VdEG1 and FoEG1, GH12 proteins with cellulase activity, are secreted from *Verticillium dahliae* and *Fusarium oxysporum*, and contribute to virulence in host plants via their enzymatic activities (Gui et al., 2017; Zhang et al., 2021). In *Botrytis cinerea*, Xyn11A, a xylanase, is required for virulence in host plants (Brito et al., 2006; Noda et al., 2010). In addition, pathogens secrete some proteins to inhibit the plant hormone‐related immune response to facilitate infection (Tariqjaveed et al., 2021). *Ustilago maydis* secretes chorismate mutase 1 (Cmu1) to eliminate the salicylic acid‐induced immune response and enhance host plant infection (Djamei et al., 2011). The powdery mildew fungus *Erysiphe quercicola* secretes the EqCSEP01276 protein, which can disturb the localization of 9‐*cis*‐epoxycarotenoid dioxygenase 5 (HbNCED5), a key enzyme for abscisic acid (ABA) biosynthesis, to suppress host immunity in plants (Li et al., 2020).

Ribonucleases (RNases), which are widely distributed in prokaryotic and eukaryotic cells and even viruses, participate in different biological activities and are classified into three families, the RNase A, RNase T1, and RNase T2 families, according to mass, base specificity, and pH preference. Secreted RNases have been reported in many pathogens and play crucial roles in plant–pathogen interactions. For example, Nuc1 and Nuc2, which are secreted from *U. maydis* and belong to the T2 family, are required for pathogenicity, and the pathogen can use extracellular RNA as a nutrient source (Mukherjee et al., 2020). Another RNase effector protein, Fg12, from *Fusarium graminearum*, contributes to virulence and possesses RNase activity to degrade the total RNA of plants (Yang et al., 2021). The RNase‐like protein CSEP0064/BEC1054 secreted from *Blumeria graminis* is structurally similar to T1 RNases and can inhibit host cell death by binding to the host ribosome to promote infection (Pennington et al., 2019). The wheat pathogen *Zymoseptoria tritici* secretes the ribonuclease protein Zt6, which possesses highly potent cytotoxic activity against wheat (Kettles et al., 2018).

*F. oxysporum* is an important soilborne fungal pathogen that has a broad host range, causes root rot and wilting disease in the plant vascular system, and results in economic loss worldwide (Zhang et al., 2021). *F. oxysporum* can infect more than 150 different plant species, including some important crops such as tomato, potato, banana, pine, and even date palm (*Phoenix canariensis*). This pathogen has many different formae speciales that can infect only one or a few host species. *F. oxysporum* can infect the vascular bundle of host plants, leading to clogged vessels, yellowing of leaves, wilting and finally death of the whole plant. The germination of dormant spores in soil results in invasion of plant roots by fungal hyphae. The movement of hyphae from root cortex to the xylem, where it produces and disseminates microconidia, is critical for disease progression. It is difficult to efficiently control this pathogen because *F. oxysporum* is soilborne and can produce chlamydospores in the soil. Chlamydospores are resistant to different environments, such as high temperature and drought. To control *F. oxysporum*, chemical fungicides are widely used. However, the resistance of this pathogen to fungicides is becoming increasingly important. To date, no effective methods to control the disease have been used in the field (McGovern, 2015).

The secreted proteins of *F. oxysporum* have been partially characterized (Li et al., 2020); however, only a small number have been functionally characterized. During the infection of tomato xylem, *F. oxysporum* secretes effectors, such as Secreted‐in‐xylem (Six) proteins, which play important roles in host specificity. Six1 is required for full virulence and is responsible for avirulence in tomato plants carrying the resistance (R) gene *I‐3*. The effector Six6 is essential for the virulence of *F. oxysporum* and suppresses host cell death (Gawehns et al., 2014). Similarly, the secreted cerato‐platanin protein FoCP1 is also required for the virulence of *F. oxysporum* on host plants (Liu et al., 2019). The M35 metalloprotease effector, another secreted protein, is also essential for full virulence (Zhang et al., 2021). In addition, FoEG1 can modulate host immunity and is required for the virulence of *F. oxysporum* (Zhang et al., 2021). However, the functions of secreted RNase proteins in *F. oxysporum* have not yet been reported.

In this study, we analysed *F. oxysporum* f. sp. *lycopersici*, a tomato pathogen, and identified the secreted RNase protein FoRnt2, which belongs to the ribonuclease T2 family and which exhibited RNase activity able to degrade the host plant total RNA. FoRnt2 was found to be required for the virulence of *F. oxysporum* in tomato seedlings. FoRnt2 also enhanced plant susceptibility to pathogens and promoted infection in plants.

## 2 RESULTS

### 2.1 FoRnt2 is a secreted protein of *F. oxysporum*

**FIGURE 1:**
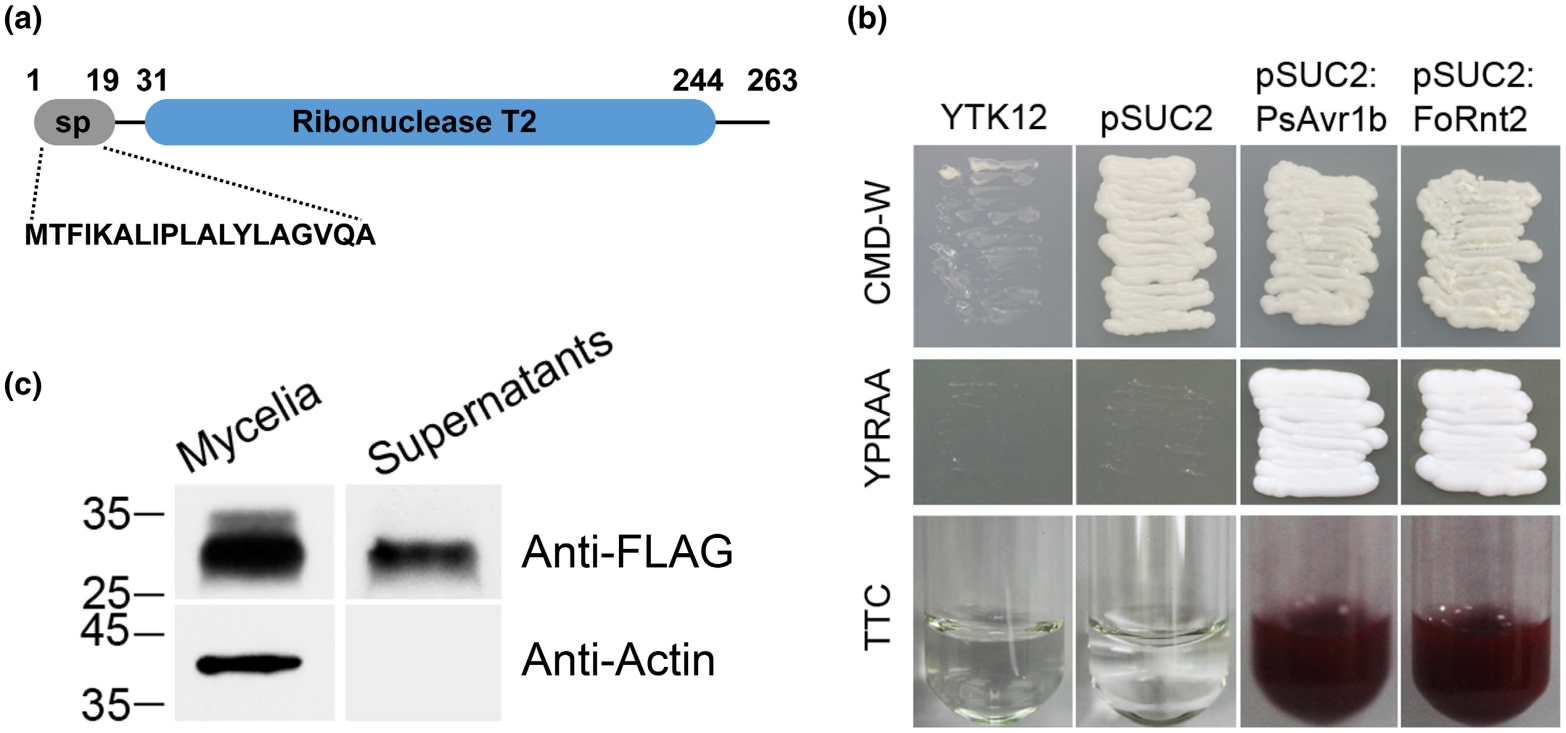
Secretion of the FoRnt2 protein from *Fusarium oxysporum*. (a) Schematic diagram of the protein domains of FoRnt2. The signal peptide (sp) was predicted using SignalP 5.0 (https://services.healthtech.dtu.dk/service.php?SignalP‐5.0). The conserved RNase domain of FoRnt2 was predicted by the SMART program (http://smart.embl‐heidelberg.de/). (b) Yeast invertase secretion assay of the predicted signal peptide of FoRnt2. CMD−W medium (lacking Trp) was used for growth of yeast strains containing the pSUC2 vector. YPRAA medium is used to indicate the yeast invertase‐enabled secretion of invertase. 2,3,triphenyltetrazolium chloride (TTC) was used to test the enzymatic activity for the reduction of TTC to red formazan. The functional signal peptide of PsAvr1b was used as the positive control in this assay. (c) An in vitro secretion assay was performed. Western blotting of total proteins in the culture supernatants induced by tomato roots and mycelia of *F. oxysporum* overexpressing FoRnt2‐FLAG were performed. Anti‐FLAG or anti‐actin were the primary antibodies used in this experiment.

We previously found an unannotated, putative secreted protein in the *F*. *oxysporum* secretome, FOXG_12372, that contained 263 amino acids (Li et al., 2020). SMART program analysis of the target protein revealed that it contained a ribonuclease T2 domain (Figure 1a), so we named it FoRnt2 (*FOXG_12372*, XP_018250940.1) in this study. In addition, the results from SignalP 5.0 predicted that FoRnt2 possesses a secretion signal peptide domain in residues 1–19 in the N‐terminal region and a cleavage site between residues 19 and 20 (Figure 1a). To validate the secretory function of the predicted signal peptide, a yeast signal trap assay was performed (Lee & Rose, 2012). The coding sequence of the N‐terminal region of FoRnt2 (amino acids 1–19) was cloned into the yeast invertase vector pSUC2, and then all the constructs were transformed into the yeast strain YTK12. The strain containing PsAvr1b was used as the positive control in this assay. All yeast strains were cultured on CMD−W plates and used to select YTK12 harbouring the pSUC2 vector. The strains containing fused FoRnt2 and PsAvr1b constructs could grow on YPRAA medium and enabled the catalysis of 2,3,5‐triphenyltetrazolium chloride (TTC) to generate the red‐coloured product triphenylformazan. In contrast, YTK12 and the strain carrying the pSUC2 vector used as a negative control did not change the colour of the culture (Figure 1b). The results confirmed that FoRnt2 has a secretory signal peptide.

To further confirm the secretory ability of the FoRnt2 protein from *F. oxysporum*, we generated the FoRnt2‐FLAG overexpression strain (Figure S1). The overexpressed strain was cultured in 10% YEPD with tomato roots that were sterilized using 75% ethanol for a secretion assay. Figure 1c shows the presence of the FoRnt2‐FLAG protein in the culture supernatant and the mycelia of the FoRnt2‐FLAG strain. Actin was detected only in the mycelia of the overexpression strain, not in the culture supernatant. This result confirmed that there was no possibility of cell lysis during mycelium‐induced growth. Taken together, the above results suggested that FoRnt2 contains a signal peptide and could be secreted from the *F. oxysporum* strain.

### 2.2 FoRnt2 has RNase activity and is highly conserved among different fungi

**FIGURE 2:**
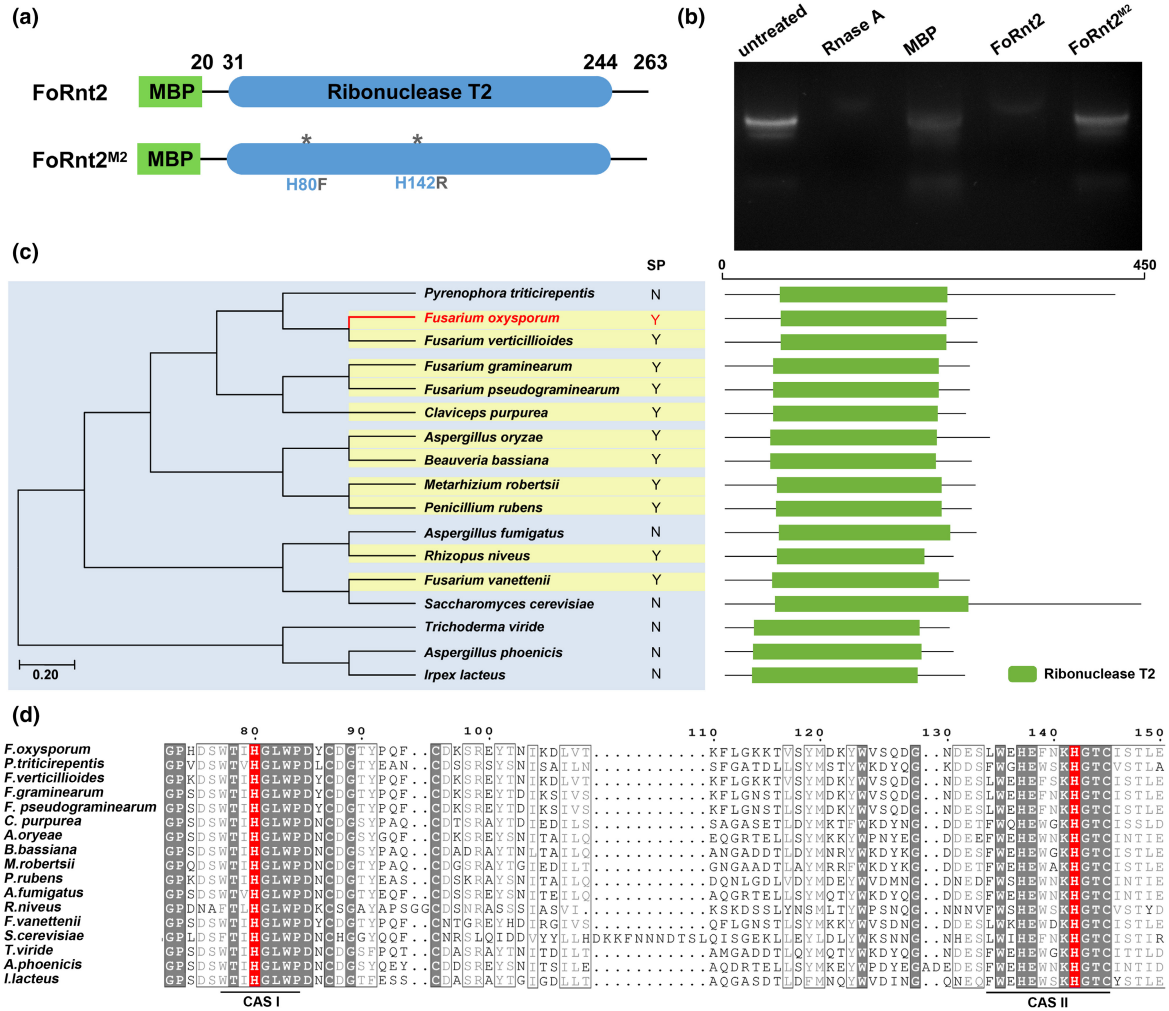
FoRnt2 possesses ribonuclease activity and is highly conserved. (a) Schematic illustration of maltose‐binding protein (MBP)‐FoRnt2 and catalytic site mutant proteins. (b) The RNase activity assay was performed using the MBP‐FoRnt2 and active site mutant MBP‐FoRnt2^M2^ proteins, which were expressed in *Escherichia coli* BL21(DE3). The tag protein MBP was used as the negative control, while RNase A was used as the positive control. Each reaction was incubated at 25°C for 30 min. (c) Phylogenetic dendrograms (neighbour joining) of FoRnt2 and other related homologous sequences from *Pyrenophora tritici‐repentis* (CAA9965927.1), *Fusarium verticillioides* (XP_018758314.1), *Fusarium graminearum* (XP_011322347.1), *Fusarium pseudograminearum* (XP_009259145.1), *Claviceps purpurea* (CCE32899.1), *Aspergillus oryzae* (P10281.2), *Beauveria bassiana* (XP_008594085.1), *Metarhizium robertsii* (XP_007816642.1), *Penicillium rubens* (XP_002556658.1), *Aspergillus fumigatus* (EDP56875.1), *Rhizopus niveus* (P08056.1), *Fusarium vanettenii* (XP_003049216.1), *Saccharomyces cerevisiae* (AHY78056.1), *Trichoderma viride* (P24657.1), *Aspergillus phoenicis* (P19791.1), and *Irpex lacteus* (AAB35880.1). Y or N represents whether the protein contained the signal peptide (SP) that was predicted using the SignalP 5.0 server. The conserved domain of each fungal protein is shown using the Pfam database prediction. (d) Protein sequence alignment of the ribonuclease T2 domain. The CAS I and CAS II regions are marked and the conserved enzyme active residues are shown in red. The protein sequence alignment was created using ESPript 5.0 (https://services.healthtech.dtu.dk/service.php?SignalP‐5.0).

The SMART program predicted that FoRnt2 possesses an RNase domain. To test the RNase activity of FoRnt2, we expressed a FoRnt2 fusion protein with a maltose‐binding protein (MBP) tag in *Escherichia coli* BL21(DE3). The two catalytic sites of the FoRnt2 fusion protein were also mutated to other amino acids, and the FoRnt2^M2^ recombinant protein was then expressed under the same conditions (Figures 2a and S2).

Next, the RNase activity of the FoRnt2 and FoRnt2^M2^ recombinant proteins was tested by incubation with tomato total RNA. FoRnt2 could significantly degrade the tomato total RNA, while the FoRnt2^M2^ proteins could not degrade the RNA under the same conditions, indicating loss of RNase activity (Figure 2b). MBP was used as a negative control, whereas RNase A was used as a positive control. These results demonstrated that FoRnt2 possessed RNase activity and that the main active site residues His80 and His142 contributed to the enzyme activity.

To explore the phylogenetic distribution of FoRnt2 homologues, we performed a protein BLAST search using the FoRnt2 protein sequence in the database to identify homologues in diverse pathogens such as *Fusarium graminearum*, *Fusarium pseudograminearum*, *Claviceps purpurea*, *Aspergillus oryzae*, *Metarhizium robertsii*, *Penicillium rubens*, *Rhizopus niveus*, and *Saccharomyces cerevisiae*. The results of phylogenetic tree analysis showed that FoRnt2 is conserved in different species, including *Beauveria bassiana*, which is a biocontrol fungus. All the proteins contained a conserved ribonuclease T2 domain that was predicted using the Pfam database according to the coding sequence (Figure 2c). In this study, we proved using different experimental methods that FoRnt2 is a secreted protein (Figure 1). To analyse whether the homologues of FoRnt2 from diverse fungi are also secreted proteins, SignalP 5.0 was used to predict the signal peptide in each protein sequence. The results revealed that most of the FoRnt2 homologues contained a predicted signal peptide, including different *Fusarium* species such as *F. graminearum* and *F. pseudograminearum*. This result indicated that FoRnt2 and most of its homologues are extracellular RNases.

Moreover, sequence alignment of the T2 family ribonuclease proteins showed that the RNase domain of FoRnt2 was similar to those of other species. An important feature of these domains is the presence of two highly conserved active site segments, termed CAS I and CASII (Figure 2d). The residues His 80 and His 142 of FoRnt2 are located in these two regions, are conserved in the homologues, and were shown to be important for RNase activity, suggesting that the two histidine residues are important for RNase function and structure in FoRnt2 and its homologues.

### 2.3 FoRnt2 is not involved in mycelial growth, various stress responses or conidiation

**FIGURE 3:**
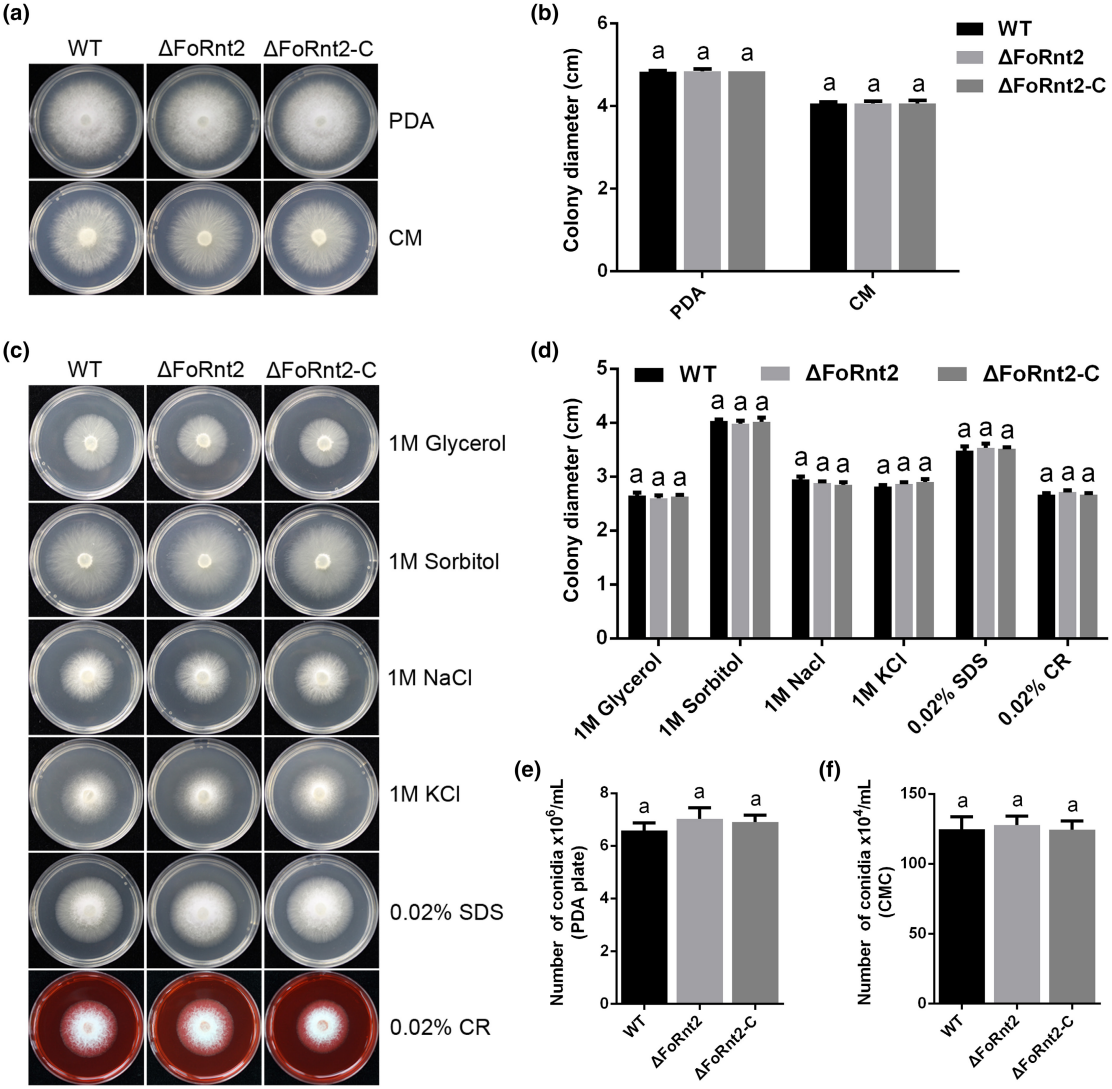
FoRnt2 is dispensable for mycelial growth, different stresses, and conidiation in *Fusarium oxysporum*. (a) Mycelial growth of the wild‐type (WT) strain, ∆FoRnt2 mutant, and ∆FoRnt2‐C complementation strain on potato dextrose agar (PDA) and complete medium (CM) for 3 days at 25°C in the dark. (b) Quantification of colony diameters for the WT strain, ∆FoRnt2 mutant, and ∆FoRnt2‐C complementation strain on PDA and CM 3 days after inoculation. (c) Mycelial growth of the indicated strains on PDA amended with 1 M glycerol, 1 M sorbitol, 1 M NaCl, 1 M KCl, 0.02% sodium dodecyl sulphate (SDS), and 0.02% Congo red (CR). All plates were cultured at 25°C for 3 days in the dark. (d) Quantification of colony diameters for all strains on PDA containing the different stress‐inducing chemicals. (e) The number of conidia of the WT, ΔFoRnt2 mutant and complementation strains were cultured on PDA for 6 days at 25°C. (f) The number of conidia of the WT, ΔFoRnt2, and complementation strains in carboxymethyl cellulose (CMC) liquid medium for 2 days. All the experiments were repeated three times. Different letters above bars indicate significant difference between mean values (*p* < 0.05, analysis of variance).

To investigate the potential function of FoRnt2, the split‐marker method was used to knock out the *FoRnt2* gene in the *F. oxysporum* wild‐type (WT) strain (Figure S3a). The *hph* gene, including the heterologous constitutive promoter, was fused with the left or right flanks of the target gene by overlap PCR (Figure S3b). The PCR products were transformed into the protoplast of the WT strain using polyethylene glycol‐mediated transformation, and the generation of the deletion mutant ∆FoRnt2 was verified by the size of the PCR products compared with those from the WT strain (Figure S3c). For complementation, the full‐length *FoRnt2* gene, including the native promoter and terminator region sequences, was transformed into the ∆FoRnt2 strain to obtain the ∆FoRnt2‐C strain, which was confirmed by PCR (Figure S3d). The growth rate and colony morphology of the ∆FoRnt2 strain were similar to those of the WT strain on potato dextrose agar (PDA) or complete medium (CM). In addition, no difference in the growth rate or colony morphology was observed between the WT strain and ∆FoRnt2‐C strain (Figure 3a,b). To test the role of FoRnt2 in mediating *F. oxysporum* adaptation to diverse stresses, we compared the growth rates of the WT, ∆FoRnt2, and ∆FoRnt2‐C strains on PDA containing the osmotic stress agents glycerol, sorbitol, NaCl, and KCl, and the cell wall‐damaging agents sodium dodecyl sulphate (SDS) and Congo red (CR). Surprisingly, we found that compared with those of the WT and ∆FoRnt2‐C strains, the growth rate of the ∆FoRnt2 strain showed no difference under any of the stresses on PDA, implying that deletion of the *FoRnt2* gene did not affect cell wall integrity or osmotic regulation (Figure 3c,d). To explore if FoRnt2 has a role in conidiation, we also measured the conidial production of all the strains on PDA and in carboxymethyl cellulose (CMC) liquid medium. Figure 3e shows that disruption of the *FoRnt2* gene did not impair conidial production of *F. oxysporum* on PDA. Similar results were also observed for the conidiation of the WT, ∆FoRnt2, and ∆FoRnt2‐C strains inoculated in CMC liquid medium (Figure 3f). These results indicate that FoRnt2 is dispensable for the conidiation of *F. oxysporum*. Taken together, the results show that FoRnt2 is not involved in mycelial growth, various stress responses or conidiation.

### 2.4 FoRnt2 is required for the virulence of *F. oxysporum*

**FIGURE 4:**
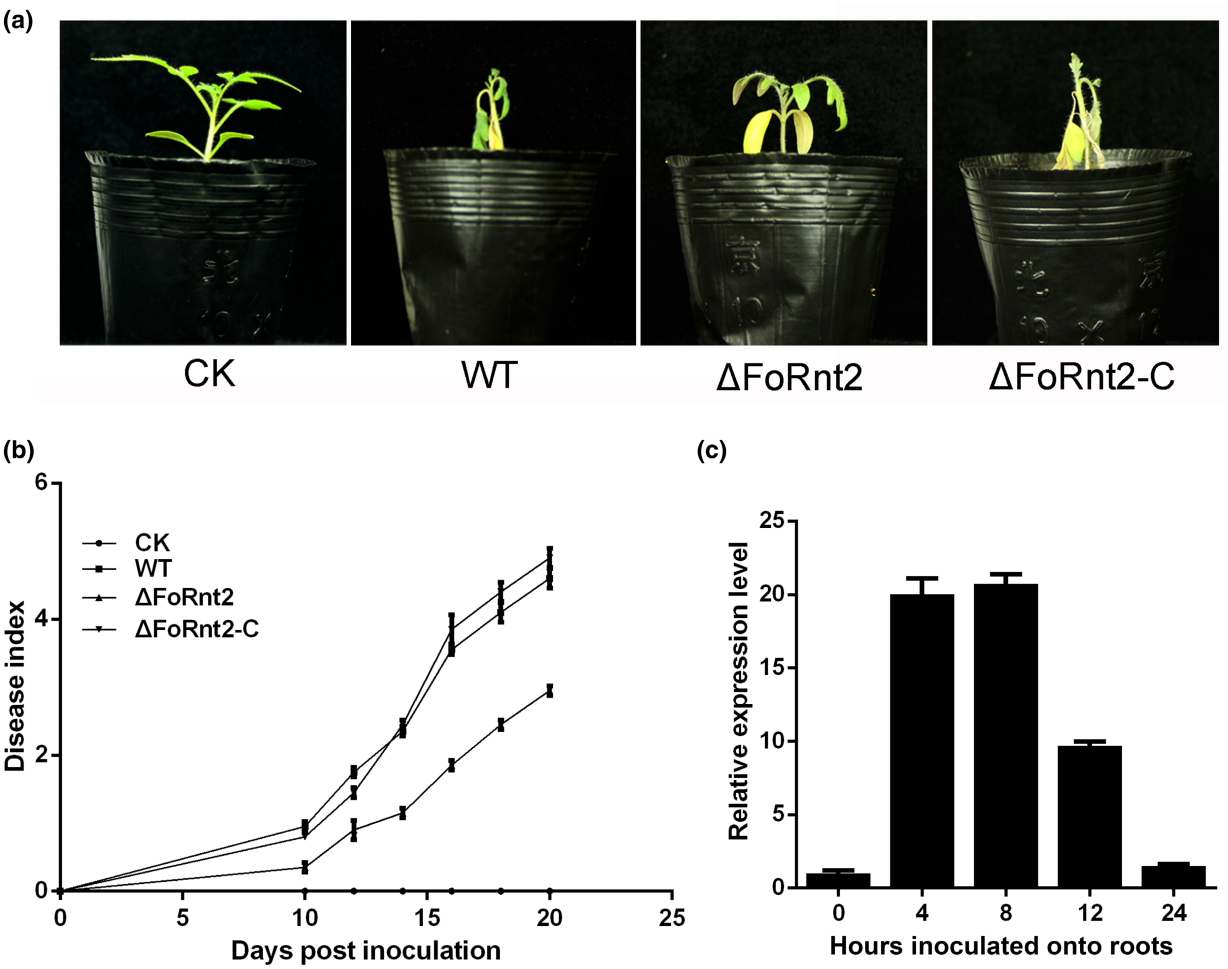
FoRnt2 is required for *Fusarium oxysporum* virulence. (a) Tomato seedlings were inoculated with the wild‐type (WT), ΔFoRnt2 mutant, and complementation strains using the root‐dip method. Photographs were taken at 20 days after infection. The infection experiments were performed three times. CK, healthy control. (b) The disease index was used to determine disease severity and was calculated using the following formula: disease index = Σ (number of plant leaves × grade value)/(total number of leaves × maximum grade value). Disease severity was defined according to a grade value from 1 to 5: 1, few symptoms, only one necrotic or curled leaf; 2, clear symptoms, three leaves exhibiting symptoms; 3, severe symptoms, necrotic and curled leaves, defoliation, growth retardation; 4, rotted plant but still alive; 5, fully dead plant. (c) The relative expression levels of *FoRnt2* were confirmed using reverse transcription‐quantitative PCR. The conidia of wild‐type *F. oxysporum* were inoculated onto tomato roots and harvested at 0, 4, 8, 12, and 24 h. The relative expression level was calibrated and set as 1 at 0 h. The constitutively expressed gene *histone 4*of *F. oxysporum* was used as an internal reference. All the experiments were repeated three times and the data are the means of three independent biological replications. The data were calculated using the 2^−∆∆*C*t^ method.

To investigate the role of FoRnt2 in *F. oxysporum* virulence, we observed the disease symptoms of tomato seedlings by using the root‐dip method. Two‐week‐old tomato seedlings were inoculated with the WT, ∆FoRnt2, and ∆FoRnt2‐C strains and then cultured for 20 days at 25°C. Typical symptoms, such as yellow leaves and growth retardation, were observed in tomato seedlings inoculated with the WT and complemented strains, while significantly reduced disease symptoms were observed in the ∆FoRnt2 strain‐infected seedlings (Figure 4a). The severity of the disease symptoms in the tomato seedlings inoculated with the WT and complemented strains increased steadily with increasing days of infection. In contrast, the plants infected by the *FoRnt2* gene deletion strain showed a significant delay in disease symptom progression compared with the WT and complemented strains (Figure 4b). Moreover, the results of reverse transcription‐quantitative real‐time PCR (RT‐qPCR) showed that the expression level of *FoRnt2* was significantly increased when conidia of *F. oxysporum* were used to inoculate tomato roots in 1% liquid YEPD medium and peaked at 8 h, showing a more than 20‐fold change (Figure 4c). These results showed that the expression level of *FoRnt2* was increased by the host plant and that deletion of the *FoRnt2* gene reduced the virulence to tomato, which indicated that FoRnt2 plays an important role in the virulence of *F. oxysporum* in the host plant.

### 2.5 FoRnt2 is localized in both the cytoplasm and nucleus in plant cells

**FIGURE 5:**
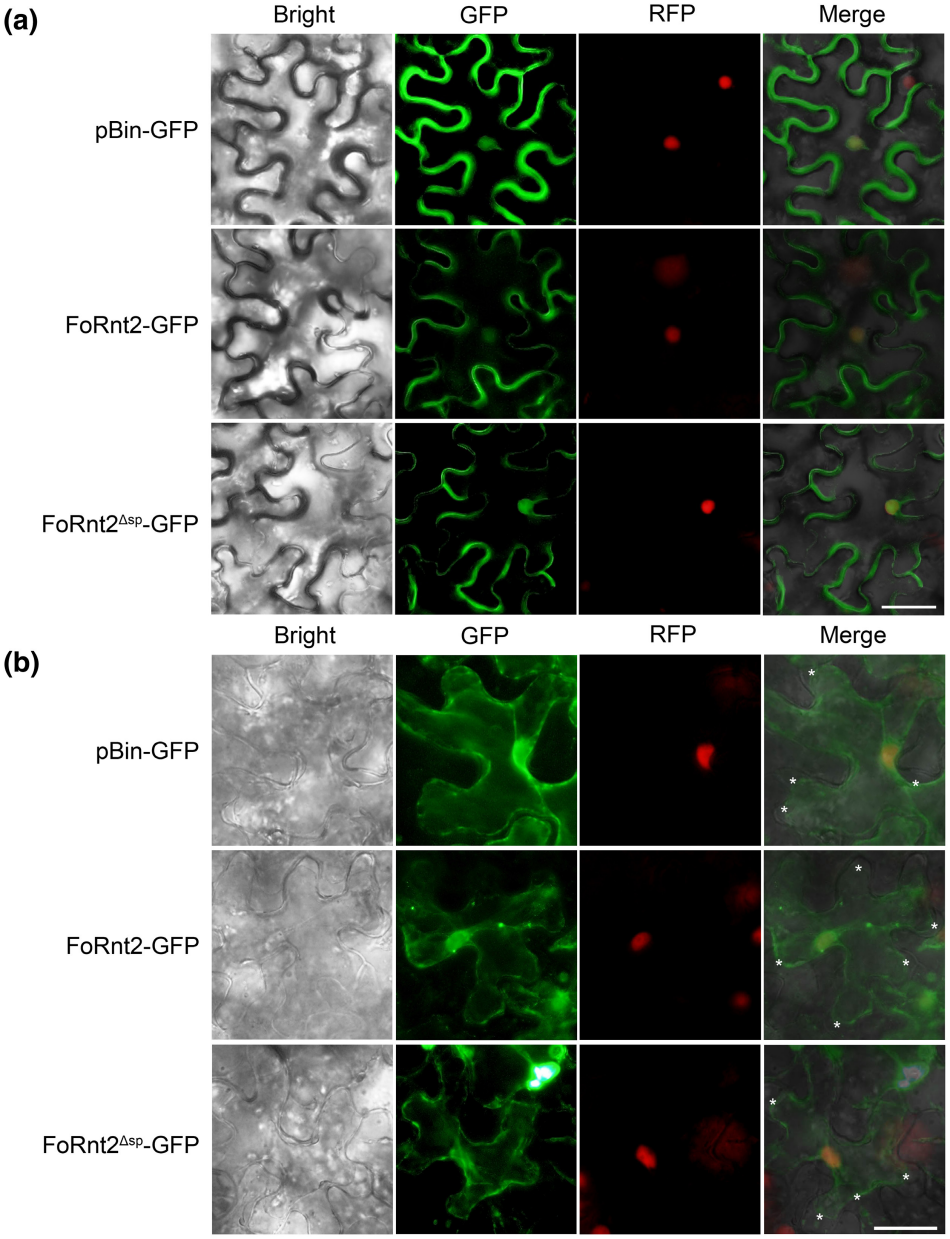
Subcellular localization of FoRnt2 in *Nicotiana benthamiana* leaves. (a) Fluorescence microscopy analysis of *N. benthamiana* leaves transiently expressing green fluorescent protein (GFP) tagged with FoRnt2 (with or without signal peptide). The images were taken at 2 days post‐agroinfiltration. pBin‐GFP was used as the negative control. H2B‐RFP (red fluorescent protein) was used to visualize the nucleus. Bars = 20 μm. (b) Fluorescence microscopy observation of *N. benthamiana* leaves after plasmolysis. GFP alone was used as the negative control. Asterisks point to the plasma membrane. Bars = 20 μm.

To detect the subcellular localization of FoRnt2 in the plant cells, we expressed green fluorescent protein (GFP) alone or FoRnt2 fused with GFP at its C‐terminus (without signal peptide) in *Nicotiana benthamiana* leaves using *Agrobacterium tumefaciens‐*mediated transient expression. Observation of the GFP distribution using microscopy was made after 2 days. GFP alone was localized in the cytoplasm and nucleus. Similarly, FoRnt2‐GFP and FoRnt2^∆sp^‐GFP were detected in the cytoplasm and nucleus. H2B‐RFP (red fluorescent protein) localizes in the nucleus and was used to verify the nuclear localization of *N. benthamiana* (Figure 5a). To further confirm FoRnt2 localization, we also observed fluorescent signals in *N. benthamiana* leaves after plasmolysis using 0.8 M NaCl. The results showed that GFP and the FoRnt2^∆sp^‐GFP and FoRnt2‐GFP fusion proteins were all localized in the cytoplasm and nucleus in *N. benthamiana* cells (Figure 5b), which further confirmed the localization of FoRnt2. In general, these findings suggest that FoRnt2 is potentially localized in the cytoplasm and nucleus of plant cells.

### 2.6 RNA‐Seq analysis of *FoRnt2* transgenic tomato

**FIGURE 6:**
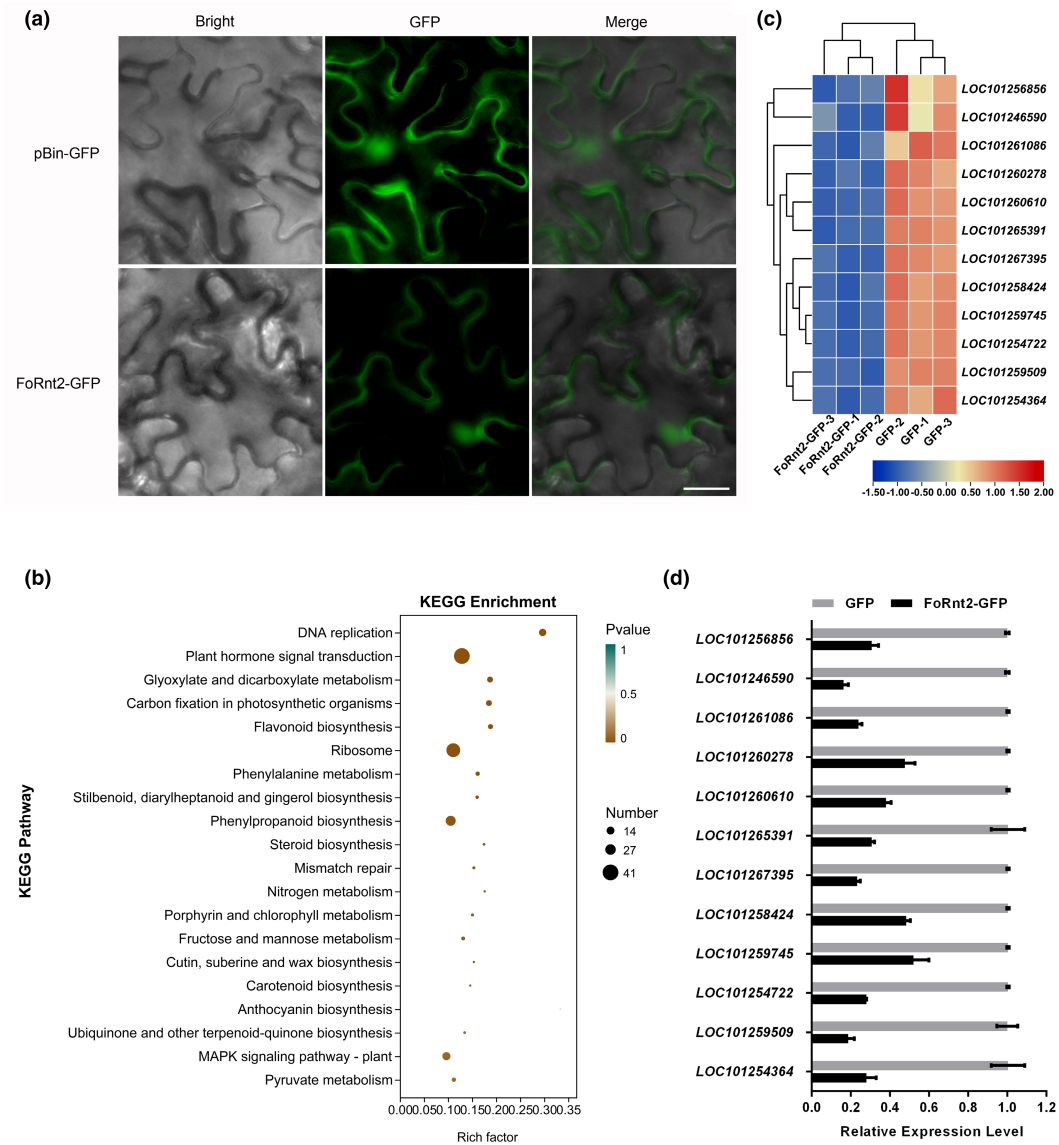
Transcriptome analysis of differentially expressed genes in *FoRnt2* transgenic tomato plants. (a) Fluorescence microscopy observation of *FoRnt2‐GFP* transgenic tomato leaves. Green fluorescent protein (GFP) alone was used as the negative control. Bars = 20 μm. (b) Top 20 pathways from KEGG functional enrichment in the down‐regulated genes. The *x* axis represents the enrichment factor. The *y* axis represents the main KEGG pathways. The point size indicates the number of target genes. The colours of the points represent the *p* values of the enriched pathways. Brown indicates high enrichment, while blue‐green indicates low enrichment. (c) Heatmap of the expression levels of candidate genes in the down‐regulated gene enriched pathways between the control (GFP) and *FoRnt2* transgenic tomato (FoRnt2‐GFP) plants. (d) Reverse transcription‐quantitative PCR analysis of the 12 down‐regulated genes in (c). The constitutively expressed gene *histone 4* of *Fusarium oxysporum* was used as an internal reference. The relative expression level of each gene was calculated using the 2^−∆∆*C*^ ^t^ method.

To further understand the potential function of FoRnt2 in transgenic tomato plants, RNA sequencing (RNA‐Seq) was used to analyse the differentially expressed genes (DEGs) in *GFP* or *FoRnt2‐GFP* transgenic tomato plants. Microscopic observation showed that green fluorescence signals were distributed in the tomato cells of the two transgenic plant lines (Figure 6a). The RNA‐Seq results identified 3340 DEGs in the GFP‐ and FoRnt2‐GFP‐expressing tomato plants, including 1877 up‐regulated genes and 1463 down‐regulated genes (Table S1 and Figure S4). This result clarified the changes in gene expression that occurred when FoRnt2 was expressed in tomato plants. To better understand the function of the down‐regulated genes, KEGG enrichment analysis was performed in this study. This showed that the down‐regulated genes were enriched in 101 pathways, mainly in information processing and metabolism (Table S2). The top 20 significantly enriched KEGG pathways, such as the DNA replication pathway, plant hormone signal transduction pathway, ribosome pathway, and nitrogen metabolism pathway, are displayed in Figure 6b. The three most enriched pathways were the plant hormone signal transduction pathway, ribosome pathway, and phenylpropanoid biosynthesis pathway, containing 41, 36, and 26 down‐regulated genes, respectively. To confirm the reliability of the expression profiles generated using RNA‐Seq and the expression pattern of down‐regulated genes, reverse transcription‐quantitative PCR (RT‐qPCR) was used. Twelve down‐regulated candidate genes in the enriched pathways were randomly selected for analysis of the expression levels in *GFP*‐ and *FoRnt2‐GFP*‐expressing tomato plants (Figure 6c). All 12 candidate genes were down‐regulated in *FoRnt2‐GFP*‐expressing tomato plants compared to *GFP*‐expressing plants. Based on the RT‐qPCR results, the expression levels of the 12 genes were correlated with the RNA‐Seq results (Figure 6d), suggesting the relative rationality and accuracy of the transcriptome analysis results in this study. These results indicated that the FoRnt2 protein plays a role in tomato plants by causing changes in the expression levels of other genes that were involved in different pathways with diverse biological functions.

### 2.7 FoRnt2 promotes fungal pathogen infection in plants

**FIGURE 7:**
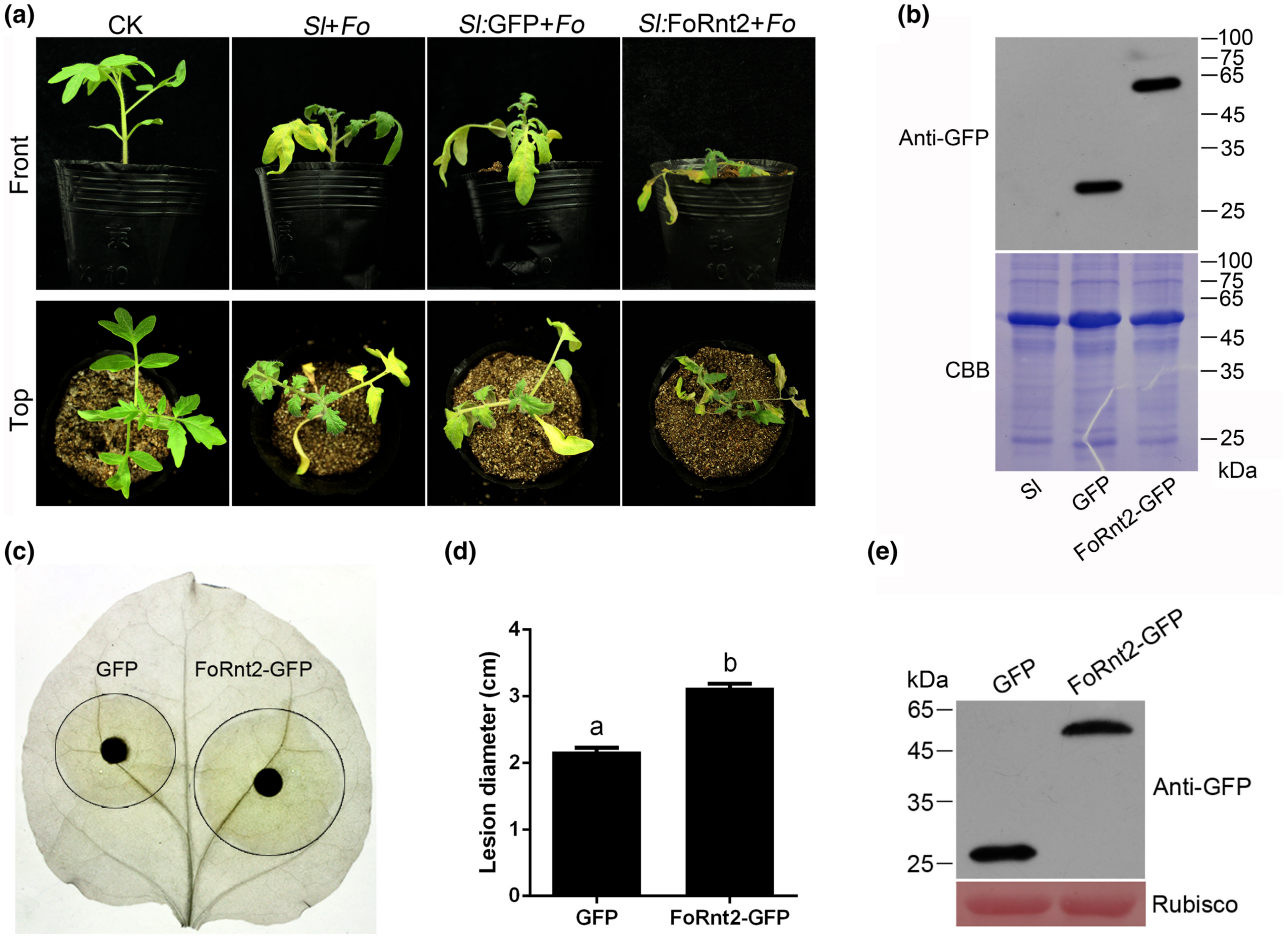
The expression of FoRnt2 promotes fungal pathogen infection in plants. (a) Wild‐type (Sl) and transgenic (Sl:GFP and Sl:FoRnt2‐GFP) tomato seedlings were inoculated with a conidial suspension of *Fusarium oxysporum* (Fo) at 5 × 10^6^ conidia/ml. The photographs were taken at 15 days postinoculation. CK, healthy control. (b) Protein expression analysis of tomato seedlings by western blotting. Coomassie brilliant blue staining (CBB) was used as a loading control to ensure that equal amounts of protein were loaded in this assay. (c) The transient expression of FoRnt2‐GFP enhances *Phytophthora capsici* infection in *Nicotiana benthamiana* leaves. The *Agrobacterium* injection area expressing GFP or FoRnt2‐GFP was inoculated with *P. capsici* at 48  h postinfiltration, and a photograph of leaves under ethanol decolourization was taken at 48 h postinoculation. (d) The lesion diameter was measured from three independent biological replicates using four leaves each time. Different letters above bars indicate significant difference (*p* < 0.05, analysis of variance). (e) The proteins of *N. benthamiana* leaves transiently expressing GFP or FoRnt2‐GFP were analysed by western blotting. Equal loading amounts of protein were confirmed by staining RuBisCO with Ponceau S.

To examine whether FoRnt2 could modulate host plant resistance to fungal pathogens, *Agrobacterium*‐mediated transgenic tomato seedlings were used in this assay. Immunoblot assays showed that GFP or FoRnt2 was expressed successfully in the stable Sl:GFP or Sl:FoRnt2 transformant plants, respectively (Figure 7b). Transgenic Sl:FoRnt2 plants showed no morphological differences compared with the transgenic Sl:GFP and wild‐type tomato plants (Figure S5), indicating that FoRnt2 has no effect in plant growth. Tomato WT seedlings and *FoRnt2* transgenic seedlings were inoculated with conidia of *F. oxysporum* using the root‐dip method. *F. oxysporum* disease symptoms were observed in plants inoculated with the pathogen after 15 days, and the findings were recorded. The Sl:FoRnt2 transgenic seedlings showed more obvious symptoms than the GFP transgenic seedlings and WT tomato (Figure 7a). The results showed that expression of FoRnt2 in tomato significantly promoted the development of disease symptoms and enhanced plant susceptibility to infection by *F. oxysporum*.

In addition, to study the potential ability of FoRnt2 to facilitate pathogen infection, we transiently expressed FoRnt2‐GFP or GFP in *Nicotiana benthamiana* using *A. tumefaciens‐*mediated transient expression. GFP was used as a negative control in this assay. Because the *F. oxysporum* strain could not infect *N. benthamiana*, *Phytophthora capsici*, a wide‐host‐range pathogen, was used to infect *N. benthamiana* leaves in this study. Two days of agroinfiltration, *P. capsici* was inoculated into *N. benthamiana* leaves expressing GFP or FoRnt2‐GFP. Forty‐eight hours after inoculation, all the leaves showed typical disease symptoms of *P. capsici* infection (Figure 7c). The lesions caused by the leaves in the area expressing FoRnt2‐GFP were significantly larger than those caused by expression of GFP alone in the *N. benthamiana* leaves (Figure 7d). Western blotting was used to detect GFP or FoRnt2‐GFP fusion protein expression in *N. benthamiana* leaves (Figure 7e). The results showed that transient expression of FoRnt2 enhanced susceptibility to pathogen infection in *N. benthamiana*. Together, these results indicated that the expression of FoRnt2 in plants could significantly promote pathogen infection.

## 3 DISCUSSION

Host–fungal pathogen interactions have been reported in many fungi, especially secreted effector proteins in filamentous pathogenic fungi, indicating the importance of effector protein function during infection (Giraldo & Valent, 2013; Hematy et al., 2009). With the development of genome sequencing and secretome identification, several potential secreted proteins have been identified in some fungi (Li et al., 2020; Lu & Edwards, 2016; Yang et al., 2021; Zhang et al., 2017). In the plant vascular pathogen *V. dahliae*, the genome data indicate that more than 700 genes are predicted to encode potential effector proteins and encode more than 200 small cysteine‐rich effectors (Chu et al., 2015; Klosterman et al., 2011; Wang et al., 2020; Zhang et al., 2017). In *Sclerotinia sclerotiorum*, a necrotrophic pathogen, SsCP1 was identified by searching the genome sequence; this protein contains a predicted signal peptide and plays an important role in virulence (Lyu et al., 2016; Yang et al., 2018; Zhang et al., 2014). Based on the genomic annotation of *F. oxysporum* strain 4287, 126 secreted proteins are encoded that are smaller than 200 amino acids in size and are cysteine rich (Ma et al., 2010). Most research to date has focused on the Six (Secreted in Xylem) proteins that were identified in the xylem during *F. oxysporum* infection of tomato (Houterman et al., 2007). In addition, some genes encoding cell wall‐degrading enzymes contribute to virulence to host plants, such as glycoside hydrolases (GHs) and pectin‐degrading enzymes, which are related to full *F. oxysporum* virulence (Bravo Ruiz et al., 2016; de Sain & Rep, 2015). In addition, RNases have been reported in the secretomes of many fungal pathogens (Brown et al., 2012; Espino et al., 2010; Yang et al., 2021). RNases hydrolyse RNA to 3′ mononucleotides via 2′,3′ cyclic nucleotides and participate in diverse biological activities (Makarov & Ilinskaya, 2003). However, there are few studies about secreted RNases involved in host‐pathogen interactions.

In this study, we found a secreted RNase protein, FoRnt2, belonging the ribonuclease T2 family in the *F. oxysporum* 4287 secretome. Similar to Nuc1 and Nuc2 of *U. maydis*, which are T2 ribonucleases, FoRnt2 can be secreted from *F. oxysporum* in the presence of its N‐terminal signal peptide (Figure 1). FoRnt2 is highly conserved in pathogenic fungi, suggesting that it may play an important role in fungal biology, such as via RNase enzyme activity. In this study, we found that FoRnt2 expressed in *E. coli* can degrade tomato total RNA in vitro. Fg12, a secreted RNase effector protein of *F. graminearum*, can also degrade the total RNA of the host plant (Yang et al., 2021). Similar results were also reported in *U. maydis*, where Nuc1 and Nuc2 degraded maize total RNA (Mukherjee et al., 2020). Enzymatic activity depends on the catalytic sites of RNase, and two conserved regions, CAS I and CASII, are observed in many fungi (Figure 2d). When the catalytic sites are mutated in the RNases of *F. graminearum* and *U. maydis*, the ability to degrade the host total RNA is lost (Mukherjee et al., 2020; Yang et al., 2021). When the key residues His 80 and His 142 of the FoRnt2 were mutated to other amino acids, FoRnt2 lost the ability to degrade RNA, indicating that the two histidine residues are crucial for RNase catalytic function (Figure 2b).

Some effector proteins are highly expressed only during invasion or on induction by the host plants. For instance, the transcript levels of many *VdSCP* genes in *V. dahliae* are up‐regulated in the presence of the host plant (Zhang et al., 2017). MoHrip1 is a secreted protein that is present in the secretome of *M. oryzae*. Up‐regulated expression of *MoHrip1* mRNA was observed in *Magnaporthe oryzae*‐infected rice (Zhang et al., 2017). Following *S. sclerotiorum* inoculation of *Arabidopsis thaliana* leaves, the transcript level of the *SsCP1* gene remains high during infection (Yang et al., 2018). The expression pattern of *FoRnt2* was first analysed using the RT‐qPCR method, and the results suggested that expression of the *FoRnt2* gene was significantly induced when *F. oxysporum* was cocultured with tomato roots, indicating that FoRnt2 may play an important role in virulence (Figure 4c). Deletion of the *FoRnt2* gene reduced the virulence of *F. oxysporum* to tomato but had no effect on mycelial growth and production of conidia (Figure 3). Other secreted RNase proteins are probably also involved in pathogen virulence, for example Fg12 is required for the full virulence of the pathogen during soybean infection, and Nuc1 and Nuc2 contribute to *U. maydis* virulence. However, the RNase effector Zt6 in *Z. tritici* is not essential for virulence to wheat (Kettles et al., 2018). It is possible that diverse secreted RNase proteins may play different roles in host–pathogen interactions. Other biological functions of RNases have been reported, such as functions in nutrition. Some RNases obtain nutrients for fungal growth by scavenging nucleotides. For instance, Nuc1 and Nuc2 in *U. maydis* are involved in nutrient acquisition through the degradation of RNAs of the host plant (Mukherjee et al., 2020). This function has been reported in many plants, especially in phosphate recycling. The T2 ribonuclease RNS2 of *Arabidopsis* is involved in the recycling of rRNA for degradation (Hillwig et al., 2011). In tomato, during phosphate starvation, RNase LX, a ribonuclease T2 protein, is involved in the RNA turnover processes in the root tip (Kock et al., 2006). Whether the FoRnt2 protein functions in nutrient acquisition for *F. oxysporum* growth or expansion still needs to be studied.

When pathogens infect host plants, the immune response is activated to block the growth and spread of pathogens. Cell death is an important mechanism of immunity in the host defence against pathogens (Kunze et al., 2004). For example, PsXGE1, a pathogen‐associated molecular pattern of *Phytophthora sojae*, strongly induces host cell death (Ma et al., 2015). SsCP1 is recognized by the PR1 protein in plants to trigger defence responses by increasing the salicylic acid content (Yang et al., 2018). RNase Fg12 strongly induces cell death in *N. benthamiana* and induces resistance to several hemibiotrophic pathogens (Yang et al., 2021). In *F. oxysporum*, FoEG1 can induce tomato and cotton cell death, and enhances host resistance to pathogens. In our study, we expressed FoRnt2 with or without its signal peptide sequence in *N. benthamiana* and cell death was not found to be induced (Figure S6). These results indicated that FoRnt2 is not like Fg12 and FoEG1 in terms of the cytotoxic effect in tobacco to induce cell death. Interestingly, FoRnt2 could promote fungal pathogen infection in plants. The *N. benthamiana* leaves expressing FoRnt2 promoted the infection of *P. capsica*, and *FoRnt2* transgenic tomato plants were more susceptible to *F. oxysporum* (Figure 7). PsCRN108, a *P. sojae* CRN effector, enhances susceptibility to *P. capsici*, while the expression of the gene in *N. benthamiana* and *A. thaliana* down‐regulates defence‐related gene expression (Song et al., 2015). Transient expression of PvRXLR11, which is an effector of *Plasmopara viticola*, in *N. benthamiana* enhances *P. capsici* infection, indicating that PvRXLR11 can suppress host defence responses by stabilizing VvWRKY40 (Ma et al., 2021). RXLR25, a virulence factor and effector of *P. capsici*, inhibits the phosphorylation of target proteins of host plants to suppress immunity and promote *Phytophthora* pathogen infection of host plants (Liang et al., 2021).

FoRnt2 promoted fungal pathogen infection in plants, indicating that FoRnt2 may suppress the defence response in the host plant under *F. oxysporum* infection. To explore the potential mechanism of interaction between host and pathogen, an RNA‐Seq analysis was performed on the *FoRnt2* transgenic plants and the control plants. The aims of the transcriptomic analysis were to investigate the differential gene expression observed when FoRnt2 was produced in the host cells and to explain how FoRnt2 promoted pathogen infection in plants. In this study, we identified 1463 down‐regulated genes. Among these, 41 down‐regulated genes were enriched in the plant hormone signal transduction pathway, most of which were related to the auxin, ethylene, and ABA signalling pathways. The positive regulation of plant defence responses against pathogens by ethylene has been documented. In *Arabidopsis*, overexpression of the ethylene response factor causes increased resistance to *B. cinerea* (Zhao et al., 2012). ABA is a positive regulator of plant defences against pathogens (Adie et al., 2007). In *N. benthamiana*, overexpression of *MeAux/IAA*s, which play an important role in the auxin signalling pathway, confer improved disease resistance against *Xanthomonas axonopodis* pv. *manihotis* infection, while the silenced plants show increased sensitivity to pathogens (Fan et al., 2020). These studies showed that some plant hormones are involved in plant resistance to pathogens. In addition, 21 down‐regulated genes were enriched in MAPK signalling pathways. Plant MAPKs play crucial roles in signalling pathways involved in plant defence against pathogens (Meng & Zhang, 2013). In the current study, FoRnt2 suppressed plant defence against pathogens, probably because FoRnt2 was transferred into the host cells and degraded RNA related to resistance to pathogens, further promoting fungal pathogen infection in the host.

The results of this study indicate that FoRnt2 has the ability to degrade host plant RNA and plays an important role in virulence. Moreover, FoRnt2 could promote fungal pathogen infection. This finding not only explains how the secreted RNase protein is important for interactions between the host and *F. oxysporum* but also further explains the pathogenic mechanism of this fungus.

## 4 EXPERIMENTAL PROCEDURES

### 4.1 Fungal strains and plants

The WT *F. oxysporum* f. sp. *lycopersici* strain 4287 (Ma et al., 2010) and its derivative strains were routinely cultured on PDA and stored at −80°C in 30% glycerine as previously described. *E. coli* DH5α and Rosetta (DE3) were cultured in lysogeny broth liquid or solid medium at 37°C for vector construction and protein expression. *A. tumefaciens* GV3101 was used to transiently express target proteins in plants. *N. benthamiana* and tomato (*Solanum lycopersicum* ‘Ailsa Craig’) were grown at 25°C under a 16 h light and 8 h dark photoperiod in an artificially controlled growth room.

### 4.2 Construction of different *F. oxysporum* strains

The split‐marker approach was used in this study to generate a gene replacement for the *FoRnt2* gene deletion mutant (Catlett et al., 2003). The PCR products were transferred into protoplasts of the WT strain as described previously. Hygromycin B (100 mg/ml) was used to select the transformants in PDA medium. The DNA of putative transformants was extracted, and then appropriate primers were used to determine whether the gene was correctly replaced. For the complementation strain, the full‐length *FoRnt2* gene, including the native promoter and terminator region sequences, was amplified and cloned into the pBS‐neo plasmid to generate the pBS‐neo‐FoRnt2 complementation vector. The coding sequence of the *FoRnt2* gene was cloned into the pHZ126‐FLAG plasmid for the overexpression vector and then transferred into protoplasts of the WT strain to obtain the FoRnt2 overexpression strain. The overexpressing strain was assayed by western blotting using anti‐FLAG antibodies (1:10000, abcam). All the transformant strains were purified by single‐spore isolation and then stored at −80°C in 30% glycerine. The primers used in the construction of different strains are listed in Table S3.

### 4.3 Yeast signal sequence trap assay to identify the signal peptides in the secreted protein

The cDNA sequences of the signal peptide regions of FoRnt2 and PsAvr1b were cloned into the pSUC2 vector, which carries a truncated invertase gene lacking both the initiation residue Met and a signal peptide (Lee & Rose, 2012; Song et al., 2015). The empty pSUC2 vector and derived vectors were transformed into yeast strain YTK12, which was grown on CMD−W medium (lacking Trp). The positive clones were transferred to YPRAA medium for the invertase secretion assay. Then, the invertase activity of all the yeast strains was determined by testing the reduction of TTC to the insoluble, red‐coloured product triphenylformazan. PsAvr1b was used as a positive control.

### 4.4 In vitro secretion assay

To test the secretion of the FoRnt2 protein, the FoRnt2‐FFLAG strain was cultured in potato dextrose broth to harvest the conidia. Then, the conidia were transferred into 10% YEPD liquid medium with the tomato roots and cultured at 25°C and 180 rpm for 16 h. The culture supernatants were collected after centrifugation. The total proteins in the culture supernatants were precipitated by adding 20% acetone (wt/vol) and stored at −80°C overnight. The solution was centrifuged at 13,800 × *g* and 4°C for 30 min. The pellets were dried and then dissolved in protein loading buffer. The target proteins were detected by western blotting using anti‐FLAG antibodies (1:10000, abcam) and anti‐actin antibodies (1:5000, abcam).

### 4.5 Agroinfiltration assays

The *FoRnt2* gene constructs were transformed into *A. tumefaciens* GV3101 through heat shock treatment. The correct *A. tumefaciens* clone was cultured overnight in LB medium (1% tryptone, 1% NaCl, and 0.5% yeast extract) at 28°C. The *A. tumefaciens* cells were collected and resuspended in infiltration buffer at an OD_600_ of 0.4. This experiment was carried out on 3‐week‐old *N. benthamiana* leaves using needleless syringes. At the same time, the same *A. tumefaciens* strain or cells harbouring the empty GFP vector were used as the negative control and BAX was used as a positive control. For subcellular localization, fluorescence observation was carried out after treatment for 48 h. For fluorescence observation after plasmolysis, the leaves of *N. benthamiana* after *Agrobacterium* injection were treated with 0.8 M NaCl under static conditions. The inoculation for *P. capsici* was performed by the same method as described previously. The total proteins in the leaves of *N. benthamiana* were extracted with protein extraction buffer and the proteins were detected by SDS‐PAGE or western blotting.

### 4.6 Plant manipulation

To generate *FoRnt2* or *GFP* transgenic tomato plants, the *FoRnt2* gene was cloned into a *GFP* construct with the CaMV 35S promoter. The resulting constructs and the 35S::GFP empty vector were transformed into WT tomato plants separately using *Agrobacterium*‐mediated transformation as described previously (Song et al., 2015). The transformant plants were first selected on medium containing 25 mg/L kanamycin and then the transformants were confirmed by fluorescence microscopy and western blotting. T_2_ transgenic lines were used for *F. oxysporum* infection.

### 4.7 Plant infection assays

To determine the role of FoRnt2 in the virulence of *F. oxysporum*, the root‐dip method of inoculation was used in this assay. Conidia of *F. oxysporum* strains were collected from potato dextrose broth cultures and then adjusted to a concentration of 5 × 10^6^ conidia/ml. Three‐week‐old tomato seedlings were inoculated with the conidial suspension or with water as a blank control for 20 min each. The infected seedlings were planted in potting soil at 25°C and 50% relative humidity with 16 h of light. The plants were observed for disease symptoms for 20 days. The disease index was recorded and calculated using a previously described method. All infection experiments were repeated three times.

### 4.8 Prokaryotic expression and purification of recombinant proteins

The coding sequence of the *FoRnt2* gene (without signal peptide) and active site mutant sequences were cloned into the expression vector pMAL‐c2x. The recombinant vectors or empty vector were transformed into *E. coli* BL21 (DE3) and the cells were cultured in LB liquid medium at 37°C. Then, the cells were diluted into fresh LB grown to a final OD of 0.6. IPTG (0.1 mM) was used to induce the expression of the recombinant proteins for 16 h at 16°C. The cells were harvested by centrifugation at 9,600 × *g* and then sonicated at 300 W for 10 min. Recombinant proteins were purified using amylose resin (BioLabs) and the protein concentration was determined using a BCA Protein Assay Kit (Solarbio). The primers used for the expression constructs are listed in Table S3.

### 4.9 Ribonuclease activity assays

The enzymatic activity of FoRnt2 was tested on total tomato RNA in an in vitro assay (Yang et al., 2021). Approximately 4 μg of total RNA was incubated with MBP (tag protein), FoRnt2 (MBP‐FoRnt2) or the active site mutant FoRnt2^M2^ (MBP‐FoRnt2^H80F/H142R^) proteins at 25°C for 30 min. Five micrograms of RNase A with equal amounts of total RNA was used as a positive control in this experiment. All samples were mixed with 1× loading buffer and then run in a 0.1% agarose gel.

### 4.10 RNA extraction and RT‐q–PCR analysis

Total RNA was extracted from the *F. oxysporum* mycelia and the plants using TRIzol reagent (Invitrogen) according the manufacturer's instructions. Reverse transcription was performed using the All‐In‐One 5× RT MasterMix Kit (Abm). HiPer SYBR Premix EsTaq (Mei5bio, 2× M5) was used for qPCR to analyse the expression pattern. The relative expression of each gene was calculated using the 2^−∆∆*C*t^ method as previously described. All experiments were repeated three times. The primers used in RT‐qPCR are listed in Table S3.

### 4.11 Bioinformatics analysis

The homologous protein sequences of FoRnt2 in different pathogens were identified by querying the FoRnt2 protein sequence against the NCBI database using the BLAST tool. The protein sequence alignment of the FoRnt2 protein and other ribonuclease T2 proteins was performed using the Clustal W2 program. Phylogenetic dendrograms of FoRnt2 and its homologous proteins were generated using MEGA 5. The signal peptides of each protein sequence were predicted by the SignalP 5.0 server (http://www.cbs.dtu.dk/services/SignalP/). The conserved domains of the proteins were identified using the Pfam database (http://pfam.xfam.org/).

### 4.12 Protein extraction

The fungal mycelia or plant tissues were ground in liquid nitrogen. The total proteins were extracted using lysis buffer (50 mM Tris–HCl [pH 8.0], 200 mM NaCl, 5% glycerol, 0.1% sodium dodecyl sulphate, 0.5 mM Triton X‐100 and 1 mM EDTA). The crude samples were centrifuged at 4°C for 15 min at 13,800 × *g*. Then, the proteins in the supernatant mixed with 10× loading buffer were incubated in a boiling water bath for 10 min. Finally, the protein samples were analysed by SDS‐PAGE.

## Supporting information


**Figure S1** Western blotting confirmation of FoRnt2‐overexpressing strains using anti‐FLAG antibodies. The targeted protein was detected in the #1 and #2 strains

**Figure S2** SDS–PAGE analysis of the FoRnt2 recombinant proteins used in this study. The proteins were expressed in *Escherichia coli* BL21 (DE3) and purified using amylose resin (BioLabs). The protein concentration was determined by a BCA kit

**Figure S3** Targeted gene knockout of *FoRnt2* in *Fusarium oxysporum*. (a) Schematic diagram of the gene deletion strategy for *FoRnt2* gene in *F. oxysporum* using the split‐marker method. (b) PCR products of each fragment and fusion amplification of split‐marker PCR. (c) Agarose gel of PCR products from the wild type (WT) and deletion strain genomic DNA. The *F. oxysporum* WT strain was used as a positive control and water was used as a negative control. M represents the molecular markers of DNA fragment size; In represents the *FoRnt2* gene; Out represents whether the *hph* gene is correctly embedded. (d) PCR confirmation of the ∆FoRnt2‐C strain. *FoRnt2* genes of the same size were detected in the WT and ∆FoRnt2‐C strains but not in the ∆FoRnt2 strain

**Figure S4** The numbers of differentially expressed genes (DEGs) in the *FoRnt2* transgenic tomato plants. The *x* axis shows the difference between green fluorescent protein (GFP) and FoRnt2‐GFP expressed in tomato. The *y* axis represents the number of DEGs. Red and blue colours represent up‐regulated and down‐regulated DEGs, respectively

**Figure S5** The morphology of 4‐week‐old green fluorescent protein (GFP) or FoRnt2‐GFP transgenic tomato and wild‐type tomato. All tomato seedlings were grown at 25°C under a 16 h light and 8 h dark photoperiod in an artificially controlled growth room

**Figure S6** FoRnt2 could not induce cell death in *Nicotiana benthamiana*. (a) The cell death‐inducing ability of FoRnt2 was determined in 3‐week‐old *N. benthamiana* leaves infiltrated with *Agrobacterium tumefaciens* carrying the target gene. BAX was used as a positive control and green fluorescent protein (GFP) was used as a negative control. The photographs were taken 4 days post‐agroinfiltration. (b) Immunoblot analysis showed the protein expression levels of GFP or GFP‐tagged protein in *N. benthamiana* leaves. Equal amounts of protein were confirmed by Ponceau S staining on the membrane

**Table S1** *FoRnt2‐GFP* The unique differentially expressed genes identified in FoRnt2‐GFP expression tomato plants

**Table S2** KEGG enrichment analysis of the down‐regulated genes in tomato plants

**Table S3** Primers used in this study

## Data Availability

The data that support the findings of this study are available from the corresponding author upon reasonable request.

## References

[mpp13237-bib-0001] Adie, B.A. , Perez‐Perez, J. , Perez‐Perez, M.M. , Godoy, M. , Sanchez‐Serrano, J.J. , Schmelz, E.A. et al. (2007) ABA is an essential signal for plant resistance to pathogens affecting JA biosynthesis and the activation of defenses in *Arabidopsis* . Plant Cell, 19, 1665–1681. 17513501 10.1105/tpc.106.048041 PMC1913739

[mpp13237-bib-0002] Blackman, L.M. , Cullerne, D.P. & Hardham, A.R. (2014) Bioinformatic characterisation of genes encoding cell wall degrading enzymes in the *Phytophthora parasitica* genome. BMC Genomics, 15, 785. 25214042 10.1186/1471-2164-15-785 PMC4176579

[mpp13237-bib-0003] Bravo Ruiz, G. , Di Pietro, A. & Roncero, M.I. (2016) Combined action of the major secreted exo‐ and endopolygalacturonases is required for full virulence of *Fusarium oxysporum* . Molecular Plant Pathology, 17, 339–353. 26060046 10.1111/mpp.12283 PMC6638378

[mpp13237-bib-0004] Brito, N. , Espino, J.J. & Gonzalez, C. (2006) The endo‐beta‐1,4‐xylanase xyn11A is required for virulence in *Botrytis cinerea* . Molecular Plant‐Microbe Interactions, 19, 25–32. 16404950 10.1094/MPMI-19-0025

[mpp13237-bib-0005] Brown, N.A. , Antoniw, J. & Hammond‐Kosack, K.E. (2012) The predicted secretome of the plant pathogenic fungus *Fusarium graminearum*: a refined comparative analysis. PLoS One, 7, e33731. 22493673 10.1371/journal.pone.0033731 PMC3320895

[mpp13237-bib-0006] Cao, L. , Blekemolen, M.C. , Tintor, N. , Cornelissen, B.J.C. & Takken, F.L.W. (2018) The *Fusarium oxysporum* Avr2‐Six5 effector pair alters plasmodesmatal exclusion selectivity to facilitate cell‐to‐cell movement of Avr2. Molecular Plant, 11, 691–705. 29481865 10.1016/j.molp.2018.02.011

[mpp13237-bib-0007] Catlett, N.L. , Lee, B.‐N. , Yoder, O.C. & Turgeon, B.G. (2003) Split‐marker recombination for efficient targeted deletion of fungal genes. Fungal Genetics Reports, 50, 9–11.

[mpp13237-bib-0008] Choi, W.S. , Lee, T.H. , Son, S.J. , Kim, T.G. , Kwon, B.M. , Son, H.U. et al. (2017) Inhibitory effect of obovatol from *Magnolia obovata* on the *Salmonella* type III secretion system. Journal of Antibiotics, 70, 1065–1069. 28874849 10.1038/ja.2017.98

[mpp13237-bib-0009] Chu, J. , Li, W.F. , Cheng, W. , Lu, M. , Zhou, K.H. , Zhu, H.Q. et al. (2015) Comparative analyses of secreted proteins from the phytopathogenic fungus *Verticillium dahliae* in response to nitrogen starvation. Biochimica et Biophysica Acta, 1854, 437–448. 25698221 10.1016/j.bbapap.2015.02.004

[mpp13237-bib-0010] Djamei, A. , Schipper, K. , Rabe, F. , Ghosh, A. , Vincon, V. , Kahnt, J. et al. (2011) Metabolic priming by a secreted fungal effector. Nature, 478, 395–398. 21976020 10.1038/nature10454

[mpp13237-bib-0011] Espino, J.J. , Gutierrez‐Sanchez, G. , Brito, N. , Shah, P. , Orlando, R. & Gonzalez, C. (2010) The *Botrytis cinerea* early secretome. Proteomics, 10, 3020–3034. 20564262 10.1002/pmic.201000037 PMC3983782

[mpp13237-bib-0012] Fan, S. , Chang, Y. , Liu, G. , Shang, S. , Tian, L. & Shi, H. (2020) Molecular functional analysis of auxin/indole‐3‐acetic acid proteins (aux/IAAs) in plant disease resistance in cassava. Physiologia Plantarum, 168, 88–97. 30950065 10.1111/ppl.12970

[mpp13237-bib-0013] Gawehns, F. , Houterman, P.M. , Ichou, F.A. , Michielse, C.B. , Hijdra, M. , Cornelissen, B.J. et al. (2014) The *Fusarium oxysporum* effector Six6 contributes to virulence and suppresses *I‐2*‐mediated cell death. Molecular Plant‐Microbe Interactions, 27, 336–348. 24313955 10.1094/MPMI-11-13-0330-R

[mpp13237-bib-0014] Giraldo, M.C. & Valent, B. (2013) Filamentous plant pathogen effectors in action. Nature Reviews Microbiology, 11, 800–814. 24129511 10.1038/nrmicro3119

[mpp13237-bib-0015] Godfrey, D. , Bohlenius, H. , Pedersen, C. , Zhang, Z. , Emmersen, J. & Thordal‐Christensen, H. (2010) Powdery mildew fungal effector candidates share N‐terminal Y/F/WxC‐motif. BMC Genomics, 11, 317. 20487537 10.1186/1471-2164-11-317 PMC2886064

[mpp13237-bib-0016] Gui, Y.J. , Chen, J.Y. , Zhang, D.D. , Li, N.Y. , Li, T.G. , Zhang, W.Q. et al. (2017) *Verticillium dahliae* manipulates plant immunity by glycoside hydrolase 12 proteins in conjunction with carbohydrate‐binding module 1. Environmental Microbiology, 19, 1914–1932. 28205292 10.1111/1462-2920.13695

[mpp13237-bib-0017] Hematy, K. , Cherk, C. & Somerville, S. (2009) Host‐pathogen warfare at the plant cell wall. Current Opinion in Plant Biology, 12, 406–413. 19616468 10.1016/j.pbi.2009.06.007

[mpp13237-bib-0018] Hillwig, M.S. , Contento, A.L. , Meyer, A. , Ebany, D. , Bassham, D.C. & Macintosh, G.C. (2011) RNS2, a conserved member of the RNase T2 family, is necessary for ribosomal RNA decay in plants. Proceedings of the National Academy of Sciences of the United States of America, 108, 1093–1098. 21199950 10.1073/pnas.1009809108 PMC3024651

[mpp13237-bib-0019] Houterman, P.M. , Speijer, D. , Dekker, H.L. , CG, D.E.K. , Cornelissen, B.J. & Rep, M. (2007) The mixed xylem sap proteome of *Fusarium oxysporum*‐infected tomato plants. Molecular Plant Pathology, 8, 215–221. 20507493 10.1111/j.1364-3703.2007.00384.x

[mpp13237-bib-0020] Ingle, R.A. , Carstens, M. & Denby, K.J. (2006) PAMP recognition and the plant‐pathogen arms race. BioEssays, 28, 880–889. 16937346 10.1002/bies.20457

[mpp13237-bib-0021] Jones, J.D. & Dangl, J.L. (2006) The plant immune system. Nature, 444, 323–329. 17108957 10.1038/nature05286

[mpp13237-bib-0022] Kettles, G.J. , Bayon, C. , Sparks, C.A. , Canning, G. , Kanyuka, K. & Rudd, J.J. (2018) Characterization of an antimicrobial and phytotoxic ribonuclease secreted by the fungal wheat pathogen *Zymoseptoria tritici* . The New Phytologist, 217, 320–331. 28895153 10.1111/nph.14786 PMC5724701

[mpp13237-bib-0023] Klosterman, S.J. , Subbarao, K.V. , Kang, S. , Veronese, P. , Gold, S.E. , Thomma, B.P. et al. (2011) Comparative genomics yields insights into niche adaptation of plant vascular wilt pathogens. PLoS Pathogens, 7, e1002137. 21829347 10.1371/journal.ppat.1002137 PMC3145793

[mpp13237-bib-0024] Kock, M. , Stenzel, I. & Zimmer, A. (2006) Tissue‐specific expression of tomato ribonuclease LX during phosphate starvation‐induced root growth. Journal of Experimental Botany, 57, 3717–3726. 16990375 10.1093/jxb/erl124

[mpp13237-bib-0025] Krause, C. , Richter, S. , Knoll, C. & Jurgens, G. (2013) Plant secretome – from cellular process to biological activity. Biochimica et Biophysica Acta, 1834, 2429–2441. 23557863 10.1016/j.bbapap.2013.03.024

[mpp13237-bib-0026] Kunze, G. , Zipfel, C. , Robatzek, S. , Niehaus, K. , Boller, T. & Felix, G. (2004) The N terminus of bacterial elongation factor Tu elicits innate immunity in *Arabidopsis* plants. The Plant Cell, 16, 3496–3507. 15548740 10.1105/tpc.104.026765 PMC535888

[mpp13237-bib-0027] Lee, S.J. & Rose, J.K. (2012) A yeast secretion trap assay for identification of secreted proteins from eukaryotic phytopathogens and their plant hosts. Methods in Molecular Biology, 835, 519–530. 22183675 10.1007/978-1-61779-501-5_32

[mpp13237-bib-0028] Li, J. , Gao, M. , Gabriel, D.W. , Liang, W. & Song, L. (2020) Secretome‐wide analysis of lysine acetylation in *Fusarium oxysporum* f. sp. lycopersici provides novel insights into infection‐related proteins. Frontiers in Microbiology, 11, 559440. 33013791 10.3389/fmicb.2020.559440 PMC7506082

[mpp13237-bib-0029] Li, X. , Liu, Y. , He, Q. , Li, S. , Liu, W. , Lin, C. et al. (2020) A candidate secreted effector protein of rubber tree powdery mildew fungus contributes to infection by regulating plant ABA biosynthesis. Frontiers in Microbiology, 11, 591387. 33324370 10.3389/fmicb.2020.591387 PMC7721678

[mpp13237-bib-0030] Liang, X. , Bao, Y. , Zhang, M. , Du, D. , Rao, S. , Li, Y. et al. (2021) A *Phytophthora capsici* RXLR effector targets and inhibits the central immune kinases to suppress plant immunity. New Phytologist, 232, 264–278. 34157161 10.1111/nph.17573

[mpp13237-bib-0031] Liu, S. , Wu, B. , Yang, J. , Bi, F. , Dong, T. , Yang, Q. et al. (2019) A cerato‐platanin family protein FocCP1 is essential for the penetration and virulence of *Fusarium oxysporum* f. sp. *cubense* tropical race 4. International Journal of Molecular Sciences, 20, 3785. 10.3390/ijms20153785 PMC6695778 31382478

[mpp13237-bib-0032] Lu, S. & Edwards, M.C. (2016) Genome‐wide analysis of small secreted cysteine‐rich proteins identifies candidate effector proteins potentially involved in *Fusarium graminearum*‐wheat interactions. Phytopathology, 106, 166–176. 26524547 10.1094/PHYTO-09-15-0215-R

[mpp13237-bib-0033] Lyu, X. , Shen, C. , Fu, Y. , Xie, J. , Jiang, D. , Li, G. et al. (2016) A small secreted virulence‐related protein is essential for the necrotrophic interactions of *Sclerotinia sclerotiorum* with its host plants. PLoS Pathogens, 12, e1005435. 26828434 10.1371/journal.ppat.1005435 PMC4735494

[mpp13237-bib-0034] Ma, L.J. , van der Does, H.C. , Borkovich, K.A. , Coleman, J.J. , Daboussi, M.J. , Di Pietro, A. et al. (2010) Comparative genomics reveals mobile pathogenicity chromosomes in *Fusarium* . Nature, 464, 367–373. 20237561 10.1038/nature08850 PMC3048781

[mpp13237-bib-0035] Ma, Z. , Song, T. , Zhu, L. , Ye, W. , Wang, Y. , Shao, Y. et al. (2015) A *Phytophthora sojae* glycoside hydrolase 12 protein is a major virulence factor during soybean infection and is recognized as a PAMP. The Plant Cell, 27, 2057–2072. 26163574 10.1105/tpc.15.00390 PMC4531360

[mpp13237-bib-0036] Ma, T. , Chen, S. , Liu, J. , Fu, P. , Wu, W. , Song, S. et al. (2021) *Plasmopara viticola* effector PvRXLR111 stabilizes VvWRKY40 to promote virulence. Molecular Plant Pathology, 22, 231–242. 33253483 10.1111/mpp.13020 PMC7814959

[mpp13237-bib-0037] Makarov, A.A. & Ilinskaya, O.N. (2003) Cytotoxic ribonucleases: molecular weapons and their targets. FEBS Letters, 540, 15–20. 12681476 10.1016/s0014-5793(03)00225-4

[mpp13237-bib-0038] McGovern, R.J. (2015) Management of tomato diseases caused by *Fusarium oxysporum* . Crop Protection, 73, 78–92.

[mpp13237-bib-0039] Meng, X. & Zhang, S. (2013) MAPK cascades in plant disease resistance signaling. Annual Review of Phytopathology, 51, 245–266. 10.1146/annurev-phyto-082712-102314 23663002

[mpp13237-bib-0040] Mengiste, T. (2012) Plant immunity to necrotrophs. Annual Review of Phytopathology, 50, 267–294. 10.1146/annurev-phyto-081211-172955 22726121

[mpp13237-bib-0041] Mukherjee, D. , Gupta, S. , Ghosh, A. & Ghosh, A. (2020) *Ustilago maydis* secreted T2 ribonucleases, Nuc1 and Nuc2 scavenge extracellular RNA. Cellular Microbiology, 22, e13256. 32844528 10.1111/cmi.13256

[mpp13237-bib-0042] Noda, J. , Brito, N. & Gonzalez, C. (2010) The *Botrytis cinerea* xylanase Xyn11A contributes to virulence with its necrotizing activity, not with its catalytic activity. BMC Plant Biology, 10, 38. 20184750 10.1186/1471-2229-10-38 PMC2844071

[mpp13237-bib-0043] Pennington, H.G. , Jones, R. , Kwon, S. , Bonciani, G. , Thieron, H. , Chandler, T. et al. (2019) The fungal ribonuclease‐like effector protein CSEP0064/BEC1054 represses plant immunity and interferes with degradation of host ribosomal RNA. PLoS Pathogens, 15, e1007620. 30856238 10.1371/journal.ppat.1007620 PMC6464244

[mpp13237-bib-0044] Postel, S. & Kemmerling, B. (2009) Plant systems for recognition of pathogen‐associated molecular patterns. Seminars in Cell & Developmental Biology, 20, 1025–1031. 19540353 10.1016/j.semcdb.2009.06.002

[mpp13237-bib-0045] Prudovsky, I. , Tarantini, F. , Landriscina, M. , Neivandt, D. , Soldi, R. , Kirov, A. et al. (2008) Secretion without Golgi. Journal of Cellular Biochemistry, 103, 1327–1343. 17786931 10.1002/jcb.21513 PMC2613191

[mpp13237-bib-0046] de Sain, M. & Rep, M. (2015) The role of pathogen‐secreted proteins in fungal vascular wilt diseases. International Journal of Molecular Sciences, 16, 23970–23993. 26473835 10.3390/ijms161023970 PMC4632733

[mpp13237-bib-0047] Sharpee, W.C. & Dean, R.A. (2016) Form and function of fungal and oomycete effectors. Fungal Biology Reviews, 30, 62–73.

[mpp13237-bib-0048] Song, T. , Ma, Z. , Shen, D. , Li, Q. , Li, W. , Su, L. et al. (2015) An oomycete CRN effector reprograms expression of plant HSP genes by targeting their promoters. PLoS Pathogens, 11, e1005348. 26714171 10.1371/journal.ppat.1005348 PMC4695088

[mpp13237-bib-0049] Tariqjaveed, M. , Mateen, A. , Wang, S. , Qiu, S. , Zheng, X. , Zhang, J. et al. (2021) Versatile effectors of phytopathogenic fungi target host immunity. Journal of Integrative Plant Biology, 63, 1856–1873. 34383388 10.1111/jipb.13162

[mpp13237-bib-0050] Wang, D. , Tian, L. , Zhang, D.D. , Song, J. , Song, S.S. , Yin, C.M. et al. (2020) Functional analyses of small secreted cysteine‐rich proteins identified candidate effectors in *Verticillium dahliae* . Molecular Plant Pathology, 21, 667–685. 32314529 10.1111/mpp.12921 PMC7170778

[mpp13237-bib-0051] Yang, B. , Wang, Q. , Jing, M. , Guo, B. , Wu, J. , Wang, H. et al. (2017) Distinct regions of the *Phytophthora* essential effector Avh238 determine its function in cell death activation and plant immunity suppression. New Phytologist, 214, 361–375. 28134441 10.1111/nph.14430

[mpp13237-bib-0052] Yang, G. , Tang, L. , Gong, Y. , Xie, J. , Fu, Y. , Jiang, D. et al. (2018) A cerato‐platanin protein SsCP1 targets plant PR1 and contributes to virulence of *Sclerotinia sclerotiorum* . New Phytologist, 217, 739–755. 29076546 10.1111/nph.14842

[mpp13237-bib-0053] Yang, B. , Wang, Y. , Tian, M. , Dai, K. , Zheng, W. , Liu, Z. et al. (2021) Fg12 ribonuclease secretion contributes to *Fusarium graminearum* virulence and induces plant cell death. Journal of Integrative Plant Biology, 63, 365–377. 32725938 10.1111/jipb.12997

[mpp13237-bib-0054] Zhang, H. , Wu, Q. , Cao, S. , Zhao, T. , Chen, L. , Zhuang, P. et al. (2014) A novel protein elicitor (SsCut) from *Sclerotinia sclerotiorum* induces multiple defense responses in plants. Plant Molecular Biology, 86, 495–511. 25149470 10.1007/s11103-014-0244-3

[mpp13237-bib-0055] Zhang, L. , Ni, H. , Du, X. , Wang, S. , Ma, X.W. , Nurnberger, T. et al. (2017) The verticillium‐specific protein VdSCP7 localizes to the plant nucleus and modulates immunity to fungal infections. New Phytologist, 215, 368–381. 28407259 10.1111/nph.14537

[mpp13237-bib-0056] Zhang, Y. , Liang, Y. , Dong, Y. , Gao, Y. , Yang, X. , Yuan, J. et al. (2017) The *Magnaporthe oryzae* Alt A 1‐like protein MoHrip1 binds to the plant plasma membrane. Biochemical and Biophysical Research Communications, 492, 55–60. 28807829 10.1016/j.bbrc.2017.08.039

[mpp13237-bib-0057] Zhang, L. , Yan, J.P. , Fu, Z.C. , Shi, W.J. , Ninkuu, V. , Li, G.Y. et al. (2021) FoEG1, a secreted glycoside hydrolase family 12 protein from *Fusarium oxysporum*, triggers cell death and modulates plant immunity. Molecular Plant Pathology, 22, 522–538. 33675158 10.1111/mpp.13041 PMC8035634

[mpp13237-bib-0058] Zhang, X. , Huang, H. , Wu, B. , Xie, J. , Viljoen, A. , Wang, W. et al. (2021) The M35 metalloprotease effector FocM35_1 is required for full virulence of *Fusarium oxysporum* f. sp. *cubense* tropical race 4. Pathogens, 10, 670. 34072465 10.3390/pathogens10060670 PMC8226822

[mpp13237-bib-0059] Zhao, Y. , Wei, T. , Yin, K.Q. , Chen, Z. , Gu, H. , Qu, L.J. et al. (2012) *Arabidopsis* RAP2.2 plays an important role in plant resistance to *Botrytis cinerea* and ethylene responses. New Phytologist, 195, 450–460. 22530619 10.1111/j.1469-8137.2012.04160.x

