# Supplementary figures and images for: The secreted ribonuclease T2 protein FoRnt2 contributes to Fusarium oxysporum virulence
Source: Mol Plant Pathol. 2022 Jun 13;23(9):1346–60. doi: 10.1111/mpp.13237 (PMC9366063; doi:10.1111/mpp.13237)

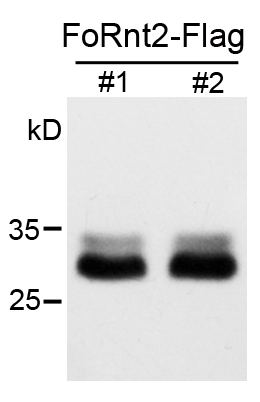

Supplement: Supplementary file 1 — Figure S1 Western blotting confirmation of FoRnt2‐overexpressing strains using anti‐FLAG antibodies. The targeted protein was detected in the #1 and #2 strains [file MPP-23-1346-s006.tif]

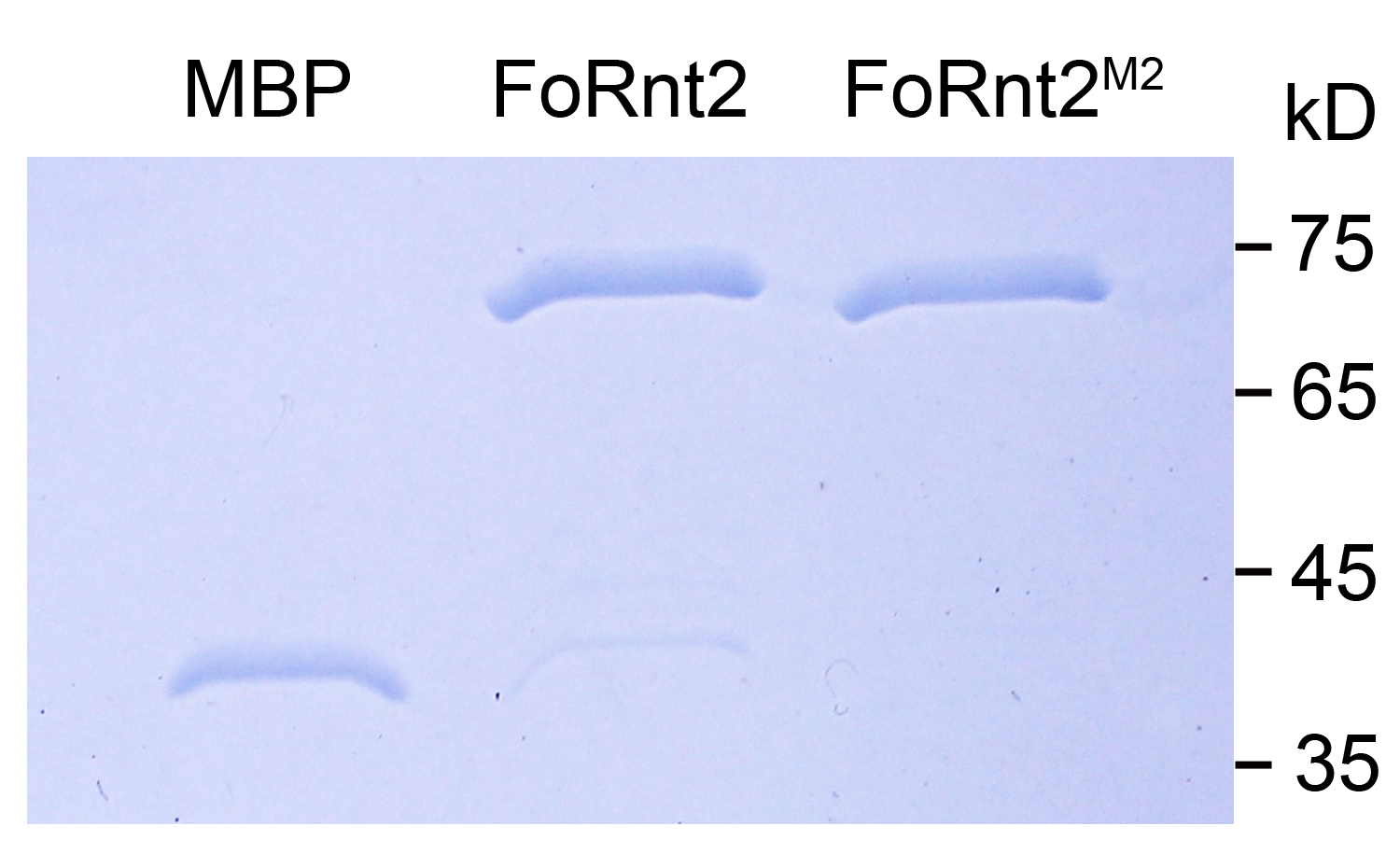

Supplement: Supplementary file 2 — Figure S2 SDS–PAGE analysis of the FoRnt2 recombinant proteins used in this study. The proteins were expressed in Escherichia coli BL21 (DE3) and purified using amylose resin (BioLabs). The protein concentration was determined by a BCA kit [file MPP-23-1346-s008.tif]

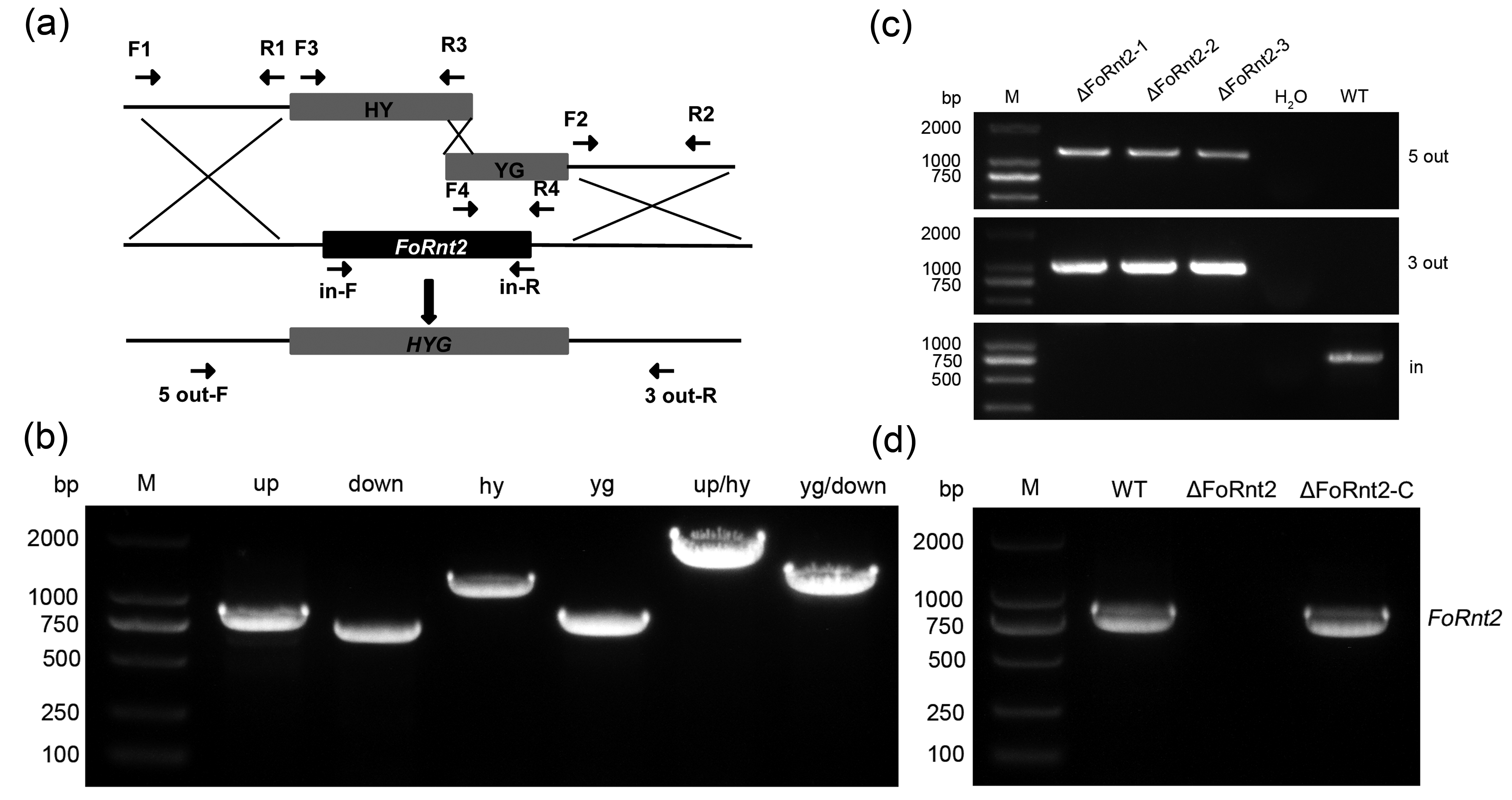

Supplement: Supplementary file 3 — Figure S3 Targeted gene knockout of FoRnt2 in Fusarium oxysporum. (a) Schematic diagram of the gene deletion strategy for FoRnt2 gene in F. oxysporum using the split‐marker method. (b) PCR products of each fragment and fusion amplification of split‐marker PCR. (c) Agarose gel of PCR products from the wild type (WT) and deletion strain genomic DNA. The F. oxysporum WT strain was used as a positive control and water was used as a negative control. M represents the molecular markers of DNA fragment size; In represents the FoRnt2 gene; Out represents whether the hph gene is correctly embedded. (d) PCR confirmation of the ∆FoRnt2‐C strain. FoRnt2 genes of the same size were detected in the WT and ∆FoRnt2‐C strains but not in the ∆FoRnt2 strain [file MPP-23-1346-s003.tif]

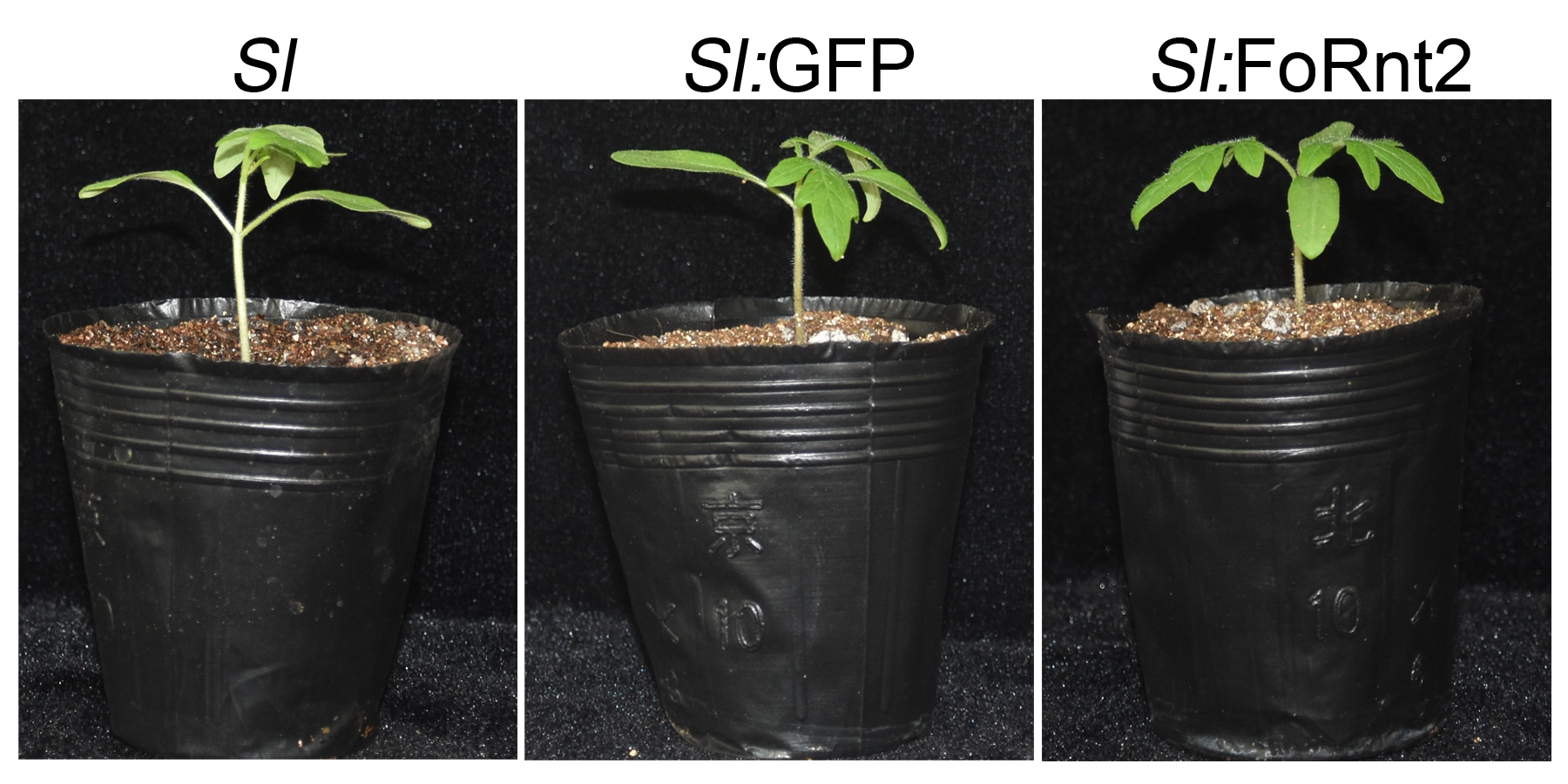

Supplement: Supplementary file 5 — Figure S5 The morphology of 4‐week‐old green fluorescent protein (GFP) or FoRnt2‐GFP transgenic tomato and wild‐type tomato. All tomato seedlings were grown at 25°C under a 16 h light and 8 h dark photoperiod in an artificially controlled growth room [file MPP-23-1346-s007.tif]

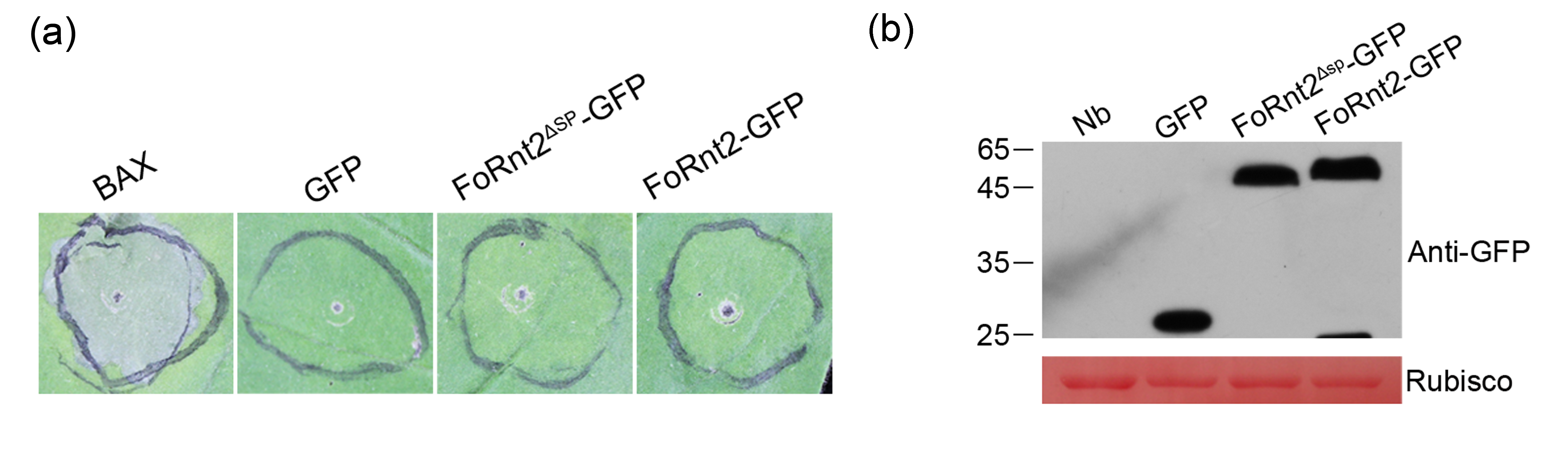

Supplement: Supplementary file 6 — Figure S6 FoRnt2 could not induce cell death in Nicotiana benthamiana. (a) The cell death‐inducing ability of FoRnt2 was determined in 3‐week‐old N. benthamiana leaves infiltrated with Agrobacterium tumefaciens carrying the target gene. BAX was used as a positive control and green fluorescent protein (GFP) was used as a negative control. The photographs were taken 4 days post‐agroinfiltration. (b) Immunoblot analysis showed the protein expression levels of GFP or GFP‐tagged protein in N. benthamiana leaves. Equal amounts of protein were confirmed by Ponceau S staining on the membrane [file MPP-23-1346-s002.tif]
