# Supplementary material for: The secreted ribonuclease T2 protein FoRnt2 contributes to Fusarium oxysporum virulence
Source: Mol Plant Pathol. 2022 Jun 13;23(9):1346–60. doi: 10.1111/mpp.13237 (PMC9366063; doi:10.1111/mpp.13237)
Supplement: Supplementary file 9 — Table S3 Primers used in this study [file MPP-23-1346-s004.docx]

**Table S3. Primers were used in this study**

| **Primer** | **Sequence (5’-3’)** | **Application** |
| --- | --- | --- |
| FoRnt2-up-F | TTGTCGACCTG CCTGACCAT | Up flank of *FoRnt2* for deletion |
| FoRnt2-up-R | GACCTCCACTAGCTCCAGCCAAGCCTAAGATTGA CCGACGAAAC CGA |  |
| FoRnt2-down-F | ATAGAGTAGATGCCGACCGCGGGTTTTAGCGCCGT GTCAACGTTG | Down flank of *FoRnt2* for deletion |
| FoRnt2-down-R | CCGACTTTT GGCTGCCAAC T |  |
| M13F | CGCCAGGGTTTTCCCAGTCACGAC | Split marker |
| M13R | AGCGGATAACAATTTCACACAGG | Split marker |
| HY | GGATGCCTCCGCTCGAAGTA | Up flank of *hph* gene with M13R |
| YG | CGTTGCAAGACCTGCCTGAA | Down flank of *hph* gene with M13F |
| FoRnt2-5-out | CAGCGTTACCACCTTGTCTG | Confirming PCR of FoRnt2 deletion mutants |
| FoRnt2-3-out | TGACGGGACTCTATCGCGAC |  |
| GC1 | ACTTCTCGACAGACGTCGC | Confirming PCR of *hph* gene |
| GC2 | TGGCTGTGTAGAAGTACTCG |  |
| FoRnt2-in-F | T TGCTGCTTCA TCCCCGCTG | Confirming PCR of *FoRnt2* gene |
| FoRnt2-in-R | GACACGAGCA CCATGGTGTT C |  |
| FoRnt2-com-F | GATATCGAATTCCTGCAGGCAGAGCTCG AACCCACGAC | Construct the *FoRnt2* complement strain |
| FoRnt2-com-R | CCACCGCGGTGGCGGCCGCCAGCTGCGCT AATTACCGAT GAG |  |
| FoRnt2-RT-F | ACCAACATCAAGGACCTCGT | Expression level of *FoRnt2* |
| FoRnt2-RT-R | TGCTCCCAGAGAGATTCGTC |  |
| H4-RT-F | CCAAGCGTCACCGAAAGATT | Expression level of *FoH4* |
| H4-RT-R | CCCTCGAGGAAGGTCTTGAG |  |
| FoRnt2-Flag-F | TTTCGTAGGAACCCAATCTTCAAA ATGACGTTCA TCAAGGCCCT C | Construct the *FoRnt2* overexpressing strain |
| FoRnt2-Flag-R | CTTTATAATCACCGTCATGGTCTTTGTAGTCCTTCTTA GGGGCATACT TGATAC |  |
| FoRnt2-H80F-F | ACTCATG GACCATTTTC GGTCTCTGGC | H80F site mutagenesis of *FoRnt2* |
| FoRnt2-H80F-R | CAGGCCAGAG ACCGAAAATG GTCCATG |  |
| FoRnt2-H142R-F | GAGTTCAAC AAGCGCGGCA CATGCATCA | H142R site mutagenesis of *FoRnt2* |
| FoRnt2-H142R-R | TGATGCATGT GCCGCGCTTG TTGAACTC |  |
| FoRnt2-psuc2-F | AATTCATGACGTTCATCAAGGCCCTCATTCCCCTTGCGCTTTACCTTGCTGGAGTGCAGGCAC | Construct pSUC2 vector for YTK12 strain |
| FoRnt2-psuc2-R | TCGAGTGCCTGCACTCCAGCAAGGTAAAGCGCAAGGGGAATGAGGGCCTTGATGAACGTCATG |  |
| pMAL-FoRnt2-F | ATTTCAGAATTCGGATCC ATG AAG TCTTGCTCTG CTGGTGG | For expression the MBP-FoRnt2 protein |
| pMAL-FoRnt2-R | GTCGACTCTAGAGGATCC TTACTTCTTA GGGGCATACT TGATAC |  |
| pQB-FoRnt2-F | ATGACGTTCA TCAAGGCCCT C | For expression the FoRnt2-GFP in plant |
| pQB-FoRnt2^Δsp^-F | ATGAAG TCTTGCTCTG CTGGTGG | For expression the FoRnt2^Δsp^-GFP in plant |
| pQB-FoRnt2-R | CTTCTTA GGGGCATACT TGATAC |  |
| LOC101261086-RT-F | ACACGCTCTTAGATGGTAGC | Expression level of *LOC101261086* in tomato |
| LOC101261086-RT-R | CGATATAGGCCGGTGTTCCA |  |
| LOC101265391-RT-F | CGCATCCCTGGCATTGATTT | Expression level of *LOC101265391* in tomato |
| LOC101265391-RT-R | TCAGCAATGGCTTACGGAGA |  |
| LOC101267395-RT-F | ATCCAGCAGCTAATGAGGCA | Expression level of *LOC101267395* in tomato |
| LOC101267395-RT-R | GGTGAGTAACCCATGTCCCA |  |
| LOC101256856-RT-F | CACGTGATCAGTTTCAGCGT | Expression level of *LOC101256856* in tomato |
| LOC101256856-RT-R | CGATTCCACCACCACTGTTC |  |
| LOC101246590-RT-F | AGGGTTTGGCTAGGTACGTT | Expression level of *LOC101246590* in tomato |
| LOC101246590-RT-R | AATTGAAGGACCTCTTGTGC |  |
| LOC101254722-RT-F | TCTTCCATTTGGGCCATCCT | Expression level of *LOC101254722* in tomato |
| LOC101254722-RT-R | CTCCAAGCTGCACTTGTGTT |  |
| LOC101259509-RT-F | AGTGCACTGTGACTTGAAGC | Expression level of *LOC101259509* in tomato |
| LOC101259509-RT-R | TGATATGCTGCTCCCGTCTT |  |
| LOC101254364-RT-F | GCATGGAGGCAGGAGAAGAT | Expression level of *LOC101254364* in tomato |
| LOC101254364-RT-R | TCTTGCGTTTGTGAATGGCA |  |
| LOC101258424-RT-F | ACTCCAATGGGACCCACATC | Expression level of *LOC101258424* in tomato |
| LOC101258424-RT-R | CACTGATGCCCTCAAACACC |  |
| LOC101259745-RT-F | AGCCTTCGACATTCAGTTGC | Expression level of *LOC101259745* in tomato |
| LOC101259745-RT-R | CGCAAGAGTTGTCATCCGTT |  |
| LOC101260278-RT-F | TTCGTGGATGCTGACAAGGA | Expression level of *LOC101260278* in tomato |
| LOC101260278-RT-R | GAGCGACCACTGAACCATTC |  |
| LOC101260610-RT-F | GACGGATGCACCACATTCAA | Expression level of *LOC101260610* in tomato |
| LOC101260610-RT-R | AGTCTTTGCGTTTGCCTTCA |  |
| SlActin-RT-F | CTGCAGGTATCCACGAGACT | Expression level of *Actin* in tomato |
| SlActin-RT-R | GCGGTGATTTCCTTGCTCAT |  |
